# Differential contributions of the proteasome, autophagy, and chaperones to the clearance of arsenite-induced protein aggregates in yeast

**DOI:** 10.1016/j.jbc.2022.102680

**Published:** 2022-11-07

**Authors:** Sansan Hua, Agnieszka Kłosowska, Joana I. Rodrigues, Gabriel Petelski, Lidia A. Esquembre, Emma Lorentzon, Lars F. Olsen, Krzysztof Liberek, Markus J. Tamás

**Affiliations:** 1 Department of Chemistry and Molecular Biology, University of Gothenburg, Göteborg, Sweden; 2 Intercollegiate Faculty of Biotechnology of University of Gdansk and Medical University of Gdansk, University of Gdansk, Gdansk, Poland; 3 Institute of Biochemistry and Molecular Biology, University of Southern Denmark, Odense M, Denmark

**Keywords:** arsenite, protein aggregation, protein degradation, ubiquitin proteasome pathway, Hsp104, Hsp70, BLI, Bio-Layer Interferometry, CHX, cycloheximide, PQC, protein quality-control, SC, synthetic complete, TBS, Tris-buffered saline, Ub, ubiquitin, UPS, ubiquitin-proteasome system

## Abstract

The poisonous metalloid arsenite induces widespread misfolding and aggregation of nascent proteins *in vivo*, and this mode of toxic action might underlie its suspected role in the pathology of certain protein misfolding diseases. Evolutionarily conserved protein quality-control systems protect cells against arsenite-mediated proteotoxicity, and herein, we systematically assessed the contribution of the ubiquitin-proteasome system, the autophagy-vacuole pathway, and chaperone-mediated disaggregation to the clearance of arsenite-induced protein aggregates in *Saccharomyces cerevisiae*. We show that the ubiquitin-proteasome system is the main pathway that clears aggregates formed during arsenite stress and that cells depend on this pathway for optimal growth. The autophagy-vacuole pathway and chaperone-mediated disaggregation both contribute to clearance, but their roles appear less prominent than the ubiquitin-proteasome system. Our *in vitro* assays with purified components of the yeast disaggregating machinery demonstrated that chaperone binding to aggregates formed in the presence of arsenite is impaired. Hsp104 and Hsp70 chaperone activity was unaffected by arsenite, suggesting that this metalloid influences aggregate structure, making them less accessible for chaperone-mediated disaggregation. We further show that the defect in chaperone-mediated refolding of a model protein was abrogated in a cysteine-free version of the substrate, suggesting that arsenite directly modifies cysteines in non-native target proteins. In conclusion, our study sheds novel light on the differential contributions of protein quality-control systems to aggregate clearance and cell proliferation and extends our understanding of how these systems operate during arsenite stress.

Cells maintain a functional proteome (protein homeostasis or proteostasis) using protein quality-control (PQC) systems that regulate protein synthesis, folding, localization, abundance, and degradation. The PQC systems include molecular chaperones that promote folding of nascent proteins into their functional conformation, help misfolded proteins to refold into their native structure, or facilitate the degradation of misfolded and damaged proteins. Cellular PQC also comprise degradation pathways such as the ubiquitin (Ub)-proteasome system (UPS) and the autophagy-lysosome system that eliminate damaged, misfolded, and aggregated proteins. The accumulation of misfolded and aggregated proteins is detrimental for cells and organisms and occurs when the activity of PQC systems decline, for example, during many age-related diseases or when cells are exposed to environmental stress that causes extensive protein misfolding, which overwhelms the PQC systems (1, 2).

Arsenic is prevalent in the environment and this toxic metalloid poses a substantial threat to human health with 100 to 200 million people estimated to be at risk (3). Exposure to arsenic can cause cancers of the skin, bladder, lung, liver, and kidney, as well as cardiovascular, respiratory, dermatological, endocrine, and neurological disorders (4, 5). Chronic exposure is also associated with neurodegenerative and age-related disorders that are characterized by the accumulation of protein aggregates, including Parkinson’s and Alzheimer’s disease (6, 7). Arsenic manifests its toxicity through multiple mechanisms. At the cellular level, arsenic interferes with redox metabolism and induces oxidative stress, impairs DNA repair mechanisms, and inhibits protein function and activity. Trivalent arsenite [As(III)] has high reactivity with sulfhydryl groups, and binding to proteins with vicinal cysteine residues can alter protein conformation, function, and interactions (5, 8, 9).

It is becoming clear that arsenic also has a profound impact on protein homeostasis and PQC (8, 10). Studies in the budding yeast *Saccharomyces cerevisiae* demonstrated that As(III) induces widespread protein misfolding and aggregation *in vivo* by targeting nascent or nonfolded proteins (11). Proteome-wide studies showed that abundant proteins with high translation rates and extensive physical interactions are primarily susceptible to aggregation during As(III) stress (12). These aggregation-prone proteins are also characterized by multiple chaperone interactions. Additionally, As(III)-aggregated protein species form seeds that increase the misfolding and aggregation of other proteins (11). Consequently, yeast cells allocate a substantial part of their genome to PQC during As(III) stress (13) and respond to As(III)-induced proteotoxicity through multiple mechanisms. Expression of genes involved in protein folding, protein degradation, As(III) chelation, and As(III) detoxification are induced while expression of genes encoding aggregation-prone proteins and protein biosynthesis–related genes are repressed (12, 14, 15, 16). In addition, cells repress translation to ensure proteostasis and cell viability during As(III) stress (13). These responses mitigate the proteotoxic effects of As(III) by restricting intracellular arsenic levels, protecting the proteome from harmful arsenic interactions, and preventing the detrimental accumulation of misfolded and aggregated proteins (11, 13, 14, 15, 17). The impact on protein homeostasis and PQC contributes to the toxicity of arsenic and may underlie its suspected role in the etiology of protein misfolding disorders (8, 10, 11, 12, 13, 18). Nevertheless, much remains to be understood about the mechanistic details of how As(III)-induced protein aggregates are formed *in vivo* and how cells regulate PQC systems to protect against toxic aggregates.

In this work, we systematically assessed the contribution of PQC systems to the clearance of As(III)-induced protein aggregates focusing on the UPS, the autophagy-vacuole pathway, and chaperone-mediated disaggregation. Our data show the differential contribution of these PQC systems to aggregate clearance and proliferation during As(III) stress where the UPS is predominant while the other pathways have auxiliary roles. We also demonstrate that chaperone binding to aggregates formed during As(III) stress is impaired, possibly due to As(III)-induced changes in aggregate structure.

## Results

### Intracellular ATP levels are maintained during As(III) exposure

**Figure 1:**
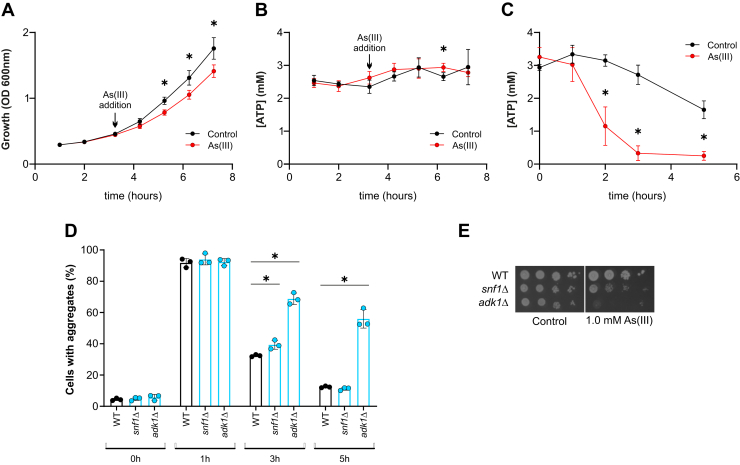
**Intracellular ATP levels are maintained during As(III) exposure.***A* and *B*, the impact of As(III) on growth (*A*) and intracellular concentration of ATP (*B*) in WT cells. Sodium arsenite to a final concentration of 0.5 mM was added to exponentially growing yeast cells as indicated by the arrow. Data are expressed as mean ± SD from three independent replicates. ∗ indicates a significant difference (*p* < 0.05) compared with cells without As(III) (unpaired, two-tailed Student’s *t* test). *C*, intracellular ATP concentration during glucose starvation. Stationary phase cells were washed and resuspended in phosphate buffer in the presence of 1 mM A(III) or an equivalent amount of NaCl (control). Data are expressed as mean ± SD from three independent replicates. ∗ indicates a significant difference (*p* < 0.05) compared with cells without As(III) (unpaired, two-tailed Student’s *t* test). *D*, Sis1–GFP distribution was scored by fluorescence microscopy in cells before and after exposure to 0.5 mM A(III). The fraction of cells containing aggregates/Sis1–GFP foci was determined by visual inspection of 98 to 190 cells per condition and time point. Data are expressed as mean ± SD from three independent biological replicates. ∗ indicates a significant difference (*p* < 0.05) compared with WT (unpaired, two-tailed Student’s *t* test). *E*, 10-fold serial dilutions of the indicated strains were placed onto agar plates with or without As(III). Growth was recorded after 2 to 3 days at 30 °C. Growth assays were performed with at least two biological replicates and a representative image is shown. OD, optical density.

Cellular ATP levels are critical for PQC because many proteins involved in protein folding and degradation require ATP hydrolysis for function (1). Moreover, a decline in ATP has been shown to be accompanied by increased protein aggregation (19); high and stable ATP levels prevent protein aggregation *in vivo* (20) and ATP by itself can keep unstructured proteins soluble (21). Thus, alterations in cellular ATP concentrations during As(III) exposure could potentially affect protein solubility and PQC. Therefore, we measured the intracellular ATP concentration in exponentially growing yeast cells exposed to As(III) using a concentration (0.5 mM) that triggers widespread protein aggregation (11, 13) but only impacts growth to a moderate extent (Fig. 1*A*). We found that the ATP levels were similar in unexposed and As(III)-exposed yeast cells (Fig. 1*B*). In a control experiment, we starved cells for glucose, a condition known to deplete cellular ATP (22), in the absence and presence of As(III). The addition of As(III) to glucose-starved cells resulted in a faster decline in ATP concentration (Fig. 1*C*). This was expected since As(III) has been shown to inhibit glycolysis in human cells by targeting hexokinase 2 (23). These results suggest that exponentially growing yeast cells maintain intracellular ATP levels during As(III) exposure.

To address whether maintaining ATP levels during As(III) stress is important for proteostasis, we used green fluorescence protein-tagged Sis1 (Sis1-GFP), an essential Hsp40 cochaperone (24) that associates with aggregation-prone proteins during proteotoxic stress (25, 26, 27), including As(III) (11, 13), as an aggregate marker. Exponentially growing cells were exposed to 0.5 mM As(III) and the percentage of cells containing Sis1-GFP foci/protein aggregates was scored. After 1 h of exposure, nearly all WT cells contained Sis1-GFP foci (Figs. 1*D* and S1*A*). The fraction of WT cells with Sis1-GFP foci/aggregates declined during sustained As(III) exposure (Figs. 1*D* and S1*A*) and this decline was approximately 2-fold faster than the increase in cell number (Fig. S1*B*), suggesting an active aggregate dissolution or clearing mechanism. It was recently shown that the AMP-activated protein kinase Snf1 and the adenylate kinase Adk1 prevent protein aggregation in unstressed yeast cells by maintaining intracellular ATP levels (20). The *adk1Δ* mutant, that has 25% to 40% lower ATP levels than the WT (20), had higher protein aggregation levels than the WT at the 3 h and 5 h time points (Fig. 1*D*), indicating defective aggregate clearance during As(III) stress. The *snf1Δ* mutant, that has 10% to 25% lower ATP levels than the WT (20), showed a minor clearance defect (Fig. 1*D*). The *adk1Δ* and *snf1Δ* mutants were As(III) sensitive, and the degree of sensitivity correlated with their ATP levels and clearance defects with *adk1Δ* showing a strong sensitivity whilst *snf1Δ* was mildly sensitive (Fig. 1*E*). Taken together, these results imply that cells maintain ATP levels during As(III) stress to ensure proteostasis. Thus, ATP-dependent PQC systems are likely to have sufficient ATP available for proper functioning during As(III) stress.

### The UPS plays a major role in the clearance of As(III)-induced protein aggregates

**Figure 2:**
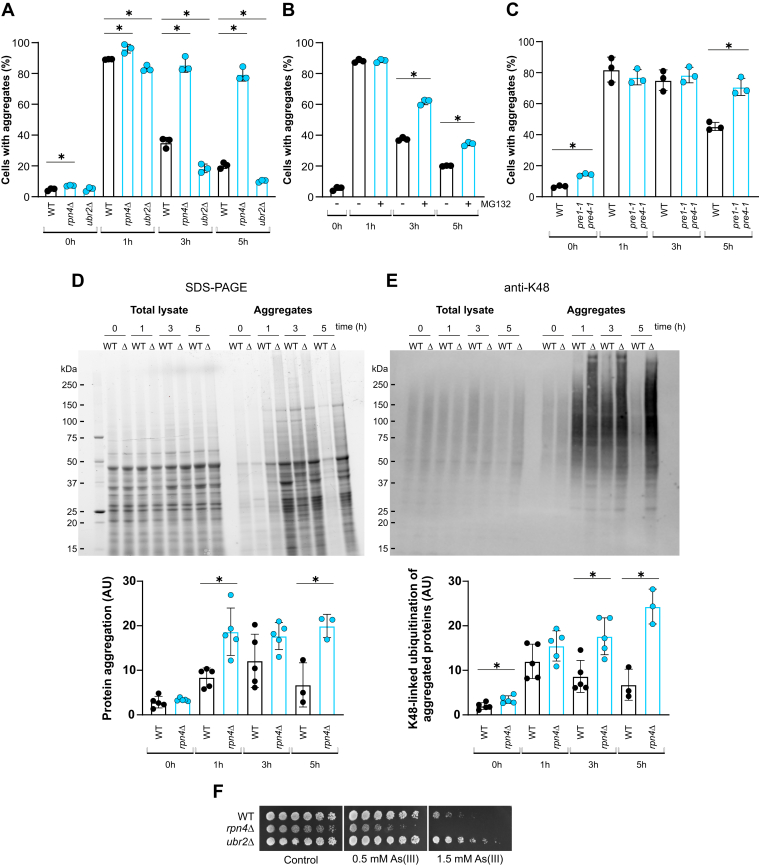
**The UPS plays a major role in the clearance of As(III)-induced protein aggregates.***A*, Hsp104–GFP distribution was scored by fluorescence microscopy in cells before and after exposure to 0.5 mM A(III). The fraction of cells containing aggregates/Hsp104–GFP foci was determined by visual inspection of 101 to 244 cells per condition and time point. Data are expressed as mean ± SD from three independent biological replicates. ∗ indicates a significant difference (*p* < 0.05) compared with WT (unpaired, two-tailed Student’s *t* test). *B*, protein aggregation (Hsp104–GFP foci) was scored as above (*A*) by visual inspection of 105 to 290 cells per condition and time point in the absence and presence of 100 μM MG132 added at the same time as 0.5 mM As(III). Data are expressed as mean ± SD from three independent biological replicates. ∗ indicates a significant difference (*p* < 0.05) compared with cells without MG132 (unpaired, two-tailed Student’s *t* test). *C*, protein aggregation (Sis1–GFP foci) was scored as aforementioned (*A*) by visual inspection of 63 to 204 cells per condition and time point before and after As(III) exposure. Data are expressed as mean ± SD from three independent biological replicates. ∗ indicates a significant difference (*p* < 0.05) compared with WT (unpaired, two-tailed Student’s *t* test). *D* and *E*, protein aggregation (*D*) and K48-linked ubiquitination (*E*). WT and *rpn4Δ* (Δ) were exposed to 0.5 mM A(III) and the proteins in the total lysate and aggregate fractions were isolated at the indicated time points, separated on SDS-PAGE and visualized as described in Experimental procedures. Immunoblotting was performed using an antibody recognizing K48-linked Ub chains. Shown is a representative gel and an immunoblot (*upper panel*) from at least three independent biological replicates. For quantification, images were analyzed using ImageJ. The signals given by the stain-free gels and the antibody from a blot were first normalized to the total signal per sample and then to the total signal over all samples. Data shown (*lower panel*) represent the average of three to five independent biological replicates with S.D. ∗ indicates a significant difference (*p* < 0.05) compared with WT (unpaired, two-tailed Student’s *t* test). *F*, 10-fold serial dilutions of the indicated strains were placed onto agar plates with or without As(III). Growth was recorded after 2 to 3 days at 30 °C. Growth assays were performed with at least two biological replicates and a representative image is shown. UPS, ubiquitin-proteasome system.

To evaluate the contribution of the UPS to aggregate clearance during As(III) stress, we used the *S. cerevisiae* disaggregase Hsp104-GFP as an aggregate marker (11, 13). As with Sis1-GFP, nearly all WT cells had Hsp104-GFP foci/protein aggregates after 1 h of exposure, whereafter the fraction of cells with aggregates declined (Figs. 2*A* and S1*A*). In contrast to WT cells, the *rpn4Δ* mutant that lacks the Rpn4 transcriptional regulator of UPS-encoding genes (28) and has reduced proteasomal activity (11), was clearly defective in clearance: the fraction of cells with aggregates was significantly higher in *rpn4Δ* than in the WT at all time points (Fig. 2*A*). Notably, after 5 h of incubation with As(III), 80% to 90% of *rpn4Δ* cells contained aggregates. Chemical inhibition of UPS activity with MG132, added at the same time as As(III), resulted in a higher fraction of cells with aggregates at the 3 h and 5 h time points (Fig. 2*B*), indicative of defective clearance. Similarly, aggregate clearance was affected in the *pre1-1 pre4-1* mutant (Fig. 2*C*) that has reduced proteasome activity due to mutations in the β-type subunits of the catalytic 20S core of the proteasome (29). Note that the WCG4 strain background of the *pre1-1 pre4-1* mutant is highly As(III) sensitive (11), explaining the slow clearance in the WCG4 WT compared to the BY4741 background used for the other experiments in this study. Thus, genetic or chemical inhibition of UPS activity results in defective clearance. In a reciprocal assay, we found that a strain lacking Ubr2 (*ubr2Δ*) that has elevated proteasomal activity due to Rpn4 stabilization (30) showed a significantly faster clearance than the WT (Fig. 2*A*).

These results suggested that the UPS plays a major role in the clearance of As(III)-induced protein aggregates, in agreement with earlier observations (11). To substantiate this, we isolated total and aggregated proteins by differential centrifugation and separated the proteins in each fraction using SDS-PAGE followed by quantification. This biochemical assay largely recapitulated the results from the Hsp104-GFP assay aforementioned: the amount of aggregated proteins first increased in response to As(III) and then declined over time in WT cells, whereas the *rpn4Δ* mutant contained more aggregated proteins compared to the WT during exposure (Fig. 2*D*). We noted that the aggregates persisted longer using the biochemical isolation method than detected by GFP-tagged chaperones: aggregate levels decreased after 3 h, as indicated by Hsp104-GFP (Fig. 2*A*) or Sis1-GFP (Fig. 1*D*) foci, whereas the decrease was evident after 5 h using the biochemical isolation method (Fig. 2*D*).

Since polyubiquitin chains involving lysine-48 (K48) in Ub signals the degradation of target proteins by the 26S proteasome (31), we subjected the samples aforementioned to immunoblotting using an antibody that specifically recognizes proteins with K48-linked Ub chains followed by signal quantification. As(III) exposure resulted in a clear increase in K48-linked ubiquitination of the proteins in the aggregate fraction (Fig. 2*E*). After an initial increase, WT cells showed a decline in aggregated proteins with K48-linked Ub chains during the timecourse of the experiment. In contrast, the amount of aggregated proteins with K48-linked ubiquitination remained high in *rpn4Δ* (Fig. 2*E*). Thus, *rpn4Δ* cells accumulate aggregated proteins with K48-linked Ub chains during As(III) stress, likely due to the lower proteasomal activity in this mutant (11).

The UPS pathway is important for optimal growth in the presence of As(III): cells with low UPS activity (*rpn4Δ* and *pre1-1 pre4-1* cells) were highly sensitive to As(III) (Fig. 2*F*) (11) whilst cells with high UPS activity (*ubr2Δ*) were clearly As(III) resistant (Fig. 2*F*).

### The autophagy-vacuole pathway plays an auxiliary role in the clearance of As(III)-induced protein aggregates

**Figure 3:**
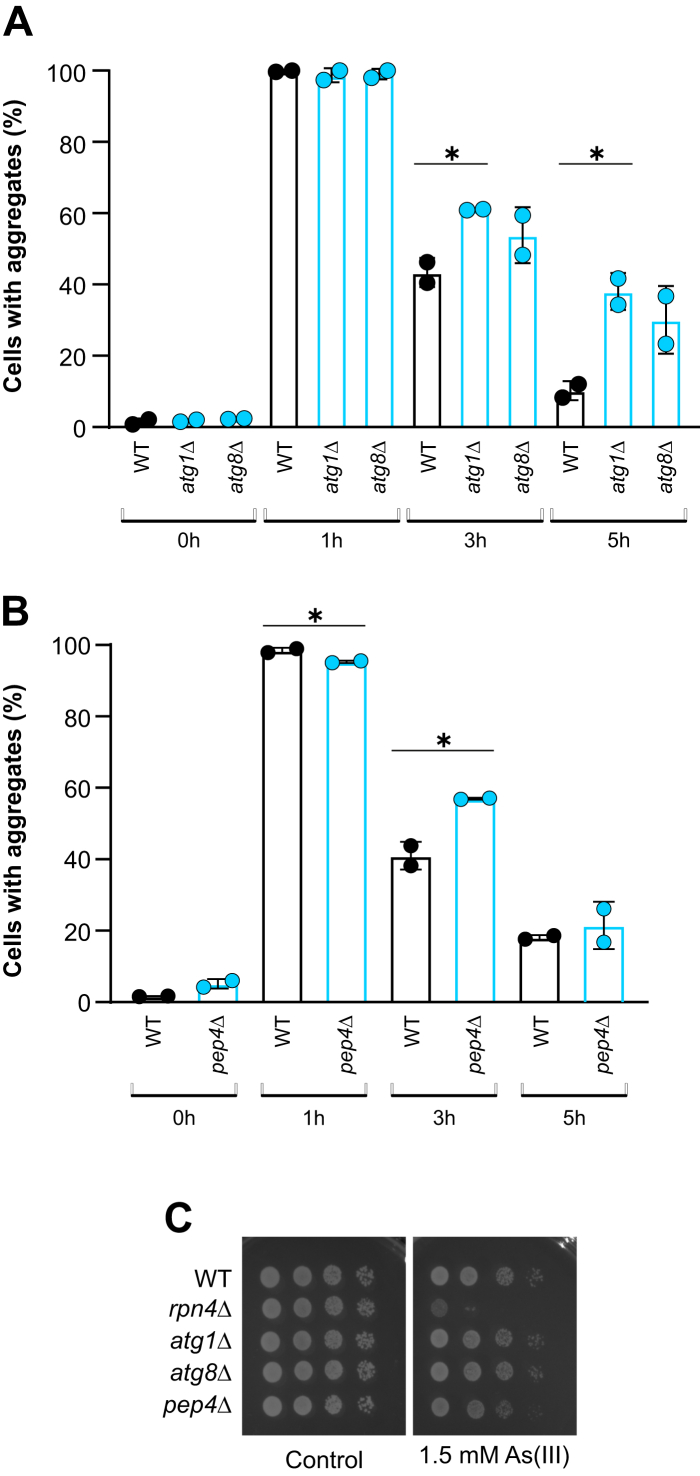
**Clearance of As(III)-induced protein aggregates involves the autophagy-vacuolar degradation pathway.***A* and *B*, Hsp104–GFP distribution was scored by fluorescence microscopy in cells before and after exposure to 0.5 mM A(III). The fraction of cells containing aggregates/Hsp104–GFP foci was determined by visual inspection of 98 to 301 cells per condition and time point. Data are expressed as mean ± SD from two independent biological replicates. ∗ indicates a significant difference (*p* < 0.05) compared with WT (unpaired, two-tailed Student’s *t* test). *C*, 10-fold serial dilutions of the indicated strains were placed onto agar plates with or without As(III). Growth was recorded after 2 to 3 days at 30 °C. Growth assays were performed with at least two biological replicates and a representative image is shown.

Besides the UPS, aggregated proteins may be targeted for degradation by the autophagy-vacuole system, and previous studies implicated this pathway in proteostasis and resistance during As(III) stress (13, 15). Indeed, mutants that lack key components of autophagy Atg1 (*atg1Δ*) and Atg8 (*atg8Δ*) (32) were less efficient in aggregate clearance compared to the WT (Fig. 3*A*). Likewise, clearance was affected in the *pep4Δ* mutant lacking the vacuolar peptidase Pep4 (33, 34) (Fig. 3*B*). Notably, the impact of *ATG1*, *ATG8*, or *PEP4* deletion on aggregate clearance was smaller than that of *RPN4* deletion. In contrast to *rpn4Δ* and *pre1-1 pre4-1* cells, As(III) did not affect growth of *atg1Δ* or *atg8Δ* whilst growth of *pep4Δ* was modestly affected (Fig. 3*C*). Additional deletion of *ATG8* in *rpn4Δ* cells (*atg8Δ rpn4Δ*) has previously been shown to exacerbate the As(III) sensitivity of *rpn4Δ* (15) pointing to a role of the autophagy-vacuole system in resistance. Altogether, these results indicate that the majority of the protein aggregates formed during As(III) exposure are marked with K48-linked Ub chains and cleared by the UPS and that this clearance pathway is critical for mitigating As(III) toxicity. The autophagy-vacuole system is less important and may have an auxiliary role.

### Chaperone-mediated disaggregation contributes to aggregate clearance during As(III) stress

**Figure 4:**
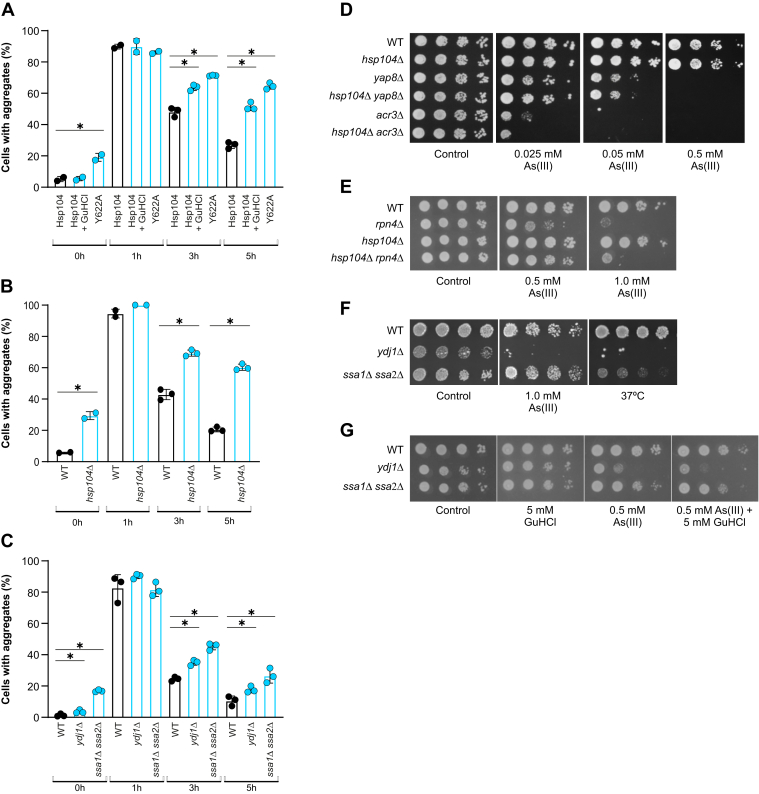
**Chaperone-mediated disaggregation contributes to aggregate clearance.***A*, Hsp104–GFP distribution was scored by fluorescence microscopy in cells before and after exposure to 0.5 mM A(III). Where indicated, Hsp104-Y662A-GFP was used or 3 mM GuHCl was added simultaneously with As(III). The fraction of cells containing aggregates/Hsp104–GFP foci was determined by visual inspection of 89 to 236 cells per condition and time point. Data are expressed as mean ± SD from at least two independent biological replicates. ∗ indicates a significant difference (*p* < 0.05) compared with the control (unpaired, two-tailed Student’s *t* test). *B*, Sis1–GFP distribution was scored by fluorescence microscopy before and after exposure to 0.5 mM As(III). The fraction of cells containing aggregates/Sis1–GFP foci was determined by visual inspection of 67 to 206 cells per condition and time point. Data are expressed as mean ± SD from at least two independent biological replicates. ∗ indicates a significant difference (*p* < 0.05) compared with WT (unpaired, two-tailed Student’s *t* test). *C*, protein aggregation (Sis1–GFP foci) was scored as aforementioned (*B*) by visual inspection of 108 to 278 cells per condition and time point. Data are expressed as mean ± SD from three independent biological replicates. ∗ indicates a significant difference (*p* < 0.05) compared with WT (unpaired, two-tailed Student’s *t* test). *D*–*G*, 10-fold serial dilutions of the indicated strains were plated onto agar plates with or without As(III). GuHCl was added as indicated. Growth was recorded after 2 to 3 days at 30 °C or at 37 °C as a control. Growth assays were performed with at least two biological replicates and a representative image is shown.

In *S. cerevisiae*, the chaperone Hsp104 (disaggregase) acts together with cytoplasmic Hsp70 (Ssa1-Ssa4) and Hsp40 cochaperones (Ydj1 or Sis1) in the disaggregation and reactivation of proteins that have misfolded and aggregated (35). To assess the role of Hsp104-mediated disaggregation in clearance, we exposed WT cells expressing Hsp104-GFP to As(III) in the absence and presence of guanidium hydrochloride (GuHCl), an inhibitor of Hsp104 activity (36, 37). The addition of GuHCl at the same time as As(III) caused a delay in aggregate clearance (Fig. 4*A*), suggesting that Hsp104 activity may be important for this process. To substantiate this, we next used a mutant version of Hsp104 (Hsp104-Y662A) that can bind to but not disassemble protein aggregates (38). Cells expressing GFP-tagged Hsp104-Y662A were defective in clearance compared to cells expressing WT Hsp104 (Fig. 4*A*). To assess clearance in the *hsp104Δ* mutant, we used Sis1-GFP as an aggregate marker. In contrast to the WT, a higher fraction of *hsp104Δ* cells accumulated Sis1-GFP foci/aggregates in the absence of As(III) and the *hsp104Δ* mutant was defective in aggregate clearance during As(III) exposure (Fig. 4*B*). Similarly, cells lacking the cytoplasmic Hsp70s Ssa1 and Ssa2 (*ssa1Δ ssa2Δ*) and the Hsp40 cochaperone Ydj1 (*ydj1Δ*) accumulated more Sis1-GFP foci/protein aggregates in the absence of stress compared to the WT and showed delayed clearance during As(III) exposure (Fig. 4*C*).

The data aforementioned implicate Hsp104-mediated disaggregation in the clearance of As(III)-induced protein aggregates. Despite this, growth of the *hsp104Δ* mutant was not affected by As(III) (Fig. 4*D*), and we asked whether the contribution of Hsp104 to resistance might be masked by the action of the major arsenic detoxification genes *YAP8* and *ACR3* (39). However, additional deletion of *HSP104* in *yap8Δ* (*hsp104Δ yap8Δ*) or *acr3Δ* (*hsp104Δ acr3Δ*) cells only marginally increased the As(III) sensitivity of the double mutants (Fig. 4*D*). Likewise, the As(III) sensitivity of the double *hsp104Δ rpn4Δ* mutant was not exacerbated compared to the single *rpn4Δ* mutant (Fig. 4*E*). Similar to *hsp104Δ*, growth of *ssa1Δ ssa2Δ* was not affected by As(III), whereas *ydj1Δ* was highly sensitive (Fig. 4*F*). Inhibition of Hsp104 by addition of GuHCl marginally increased the As(III) sensitivity of *ydj1Δ* and *ssa1Δ ssa2Δ* cells (Fig. 4*G*). We conclude that Hsp104-dependent disaggregation contributes to aggregate clearance but is largely dispensable for growth during As(III) stress.

### Hsp104 overexpression is toxic during As(III) stress

**Figure 5:**
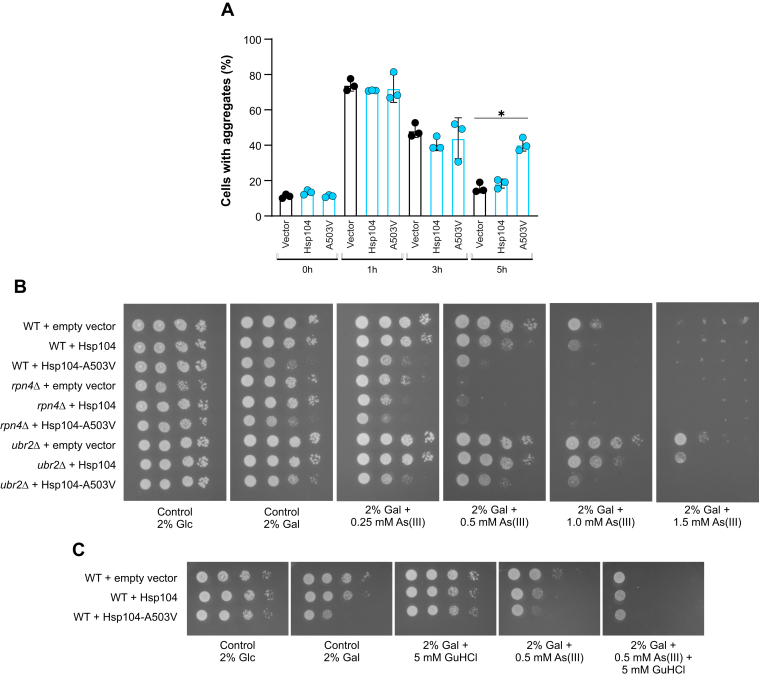
**Hsp104 overexpression is toxic during As(III) stress.***A*, cells harboring a genomic copy of Hsp104-GFP and the indicated plasmids were grown on galactose for 3 h to induce expression of the indicated plasmid-encoded genes. Hsp104–GFP distribution was scored by fluorescence microscopy before and after exposure to 0.5 mM As(III). The fraction of cells containing aggregates/Hsp104-GFP foci was determined by visual inspection of 123 to 339 cells per condition and time point. Data are expressed as mean ± SD from three independent biological replicates. ∗ indicates a significant difference (*p* < 0.05) compared with WT (unpaired, two-tailed Student’s *t* test). *B*, 10-fold serial dilutions of cells harboring a genomic copy of Hsp104-GFP and the indicated plasmids were plated onto agar plates with or without As(III). The presence of glucose (Glc) keeps expression from the *GAL1* promoter off whilst the presence of galactose (Gal) induces expression of Hsp104 and Hsp104-A503V. Growth was recorded after 2 to 3 days at 30 °C. Growth assays were performed with at least two biological replicates and a representative image is shown. *C*, cells were grown to log phase in medium containing raffinose and 10-fold serial dilutions of the cultures were plated on agar plates with or without glucose (Glc), galactose (Gal), As(III), or GuHCl. Growth was monitored after 2 to 3 days at 30 °C. Growth assays were performed with three biological replicates and a representative image is shown.

Since Hsp104 contributes to clearance, we asked whether an increase in Hsp104 activity would be beneficial for aggregate clearance and As(III) resistance. For this, we overexpressed Hsp104 from the inducible *GAL1* promoter in WT cells harboring a genomic copy of Hsp104-GFP. We also expressed a so-called ‘potentiated’ version of Hsp104 (Hsp104-A503V) that has elevated ATPase, disaggregase, and unfoldase activities (40). Overexpression of WT Hsp104 did not affect aggregate clearance during As(III) exposure, whereas expression of Hsp104-A503V resulted in less efficient clearance (Fig. 5*A*). This finding was unexpected given the fact that Hsp104-A503V mitigates the aggregation and toxicity of the neurodegenerative disease–associated proteins α-synuclein and FUS in yeast (40).

Growth assays showed that overexpression of Hsp104 sensitized cells to As(III) (Fig. 5*B*). Similarly, overexpression of Hsp104-A503V was toxic to cells (40) and the presence of As(III) aggravated its toxicity (Fig. 5*B*). To address whether the observed toxicity is dependent on Hsp104 activity, we performed the same growth assays in the presence of GuHCl. Indeed, the addition of GuHCl clearly mitigated the toxicity caused by Hsp104 and Hsp104-A503V overexpression, both in the absence and presence of As(III) (Fig. 5*C*). These findings suggest that a tight control of Hsp104 activity is important for optimal growth during As(III) stress.

To test whether Hsp104-induced toxicity is affected by the UPS, we scored growth of *rpn4Δ* (low proteasomal activity) and *ubr2Δ* (high proteasomal activity) cells overexpressing Hsp104 or Hsp104-A503V. Albeit being As(III) sensitive, *rpn4Δ* cells were similarly affected by Hsp104 or Hsp104-A503V overexpression as the WT. Likewise, the As(III) resistant *ubr2Δ* mutant was similarly affected by Hsp104 or Hsp104-A503V overexpression as the WT (Fig. 5*B*). The absence of phenotypic exacerbation or improvement in the *rpn4Δ* and *ubr2Δ* backgrounds, respectively, suggests that Hsp104-mediated toxicity is independent of the UPS. We speculate that high protein-unfolding activity is detrimental during As(III) stress, possibly by the accumulation of misfolded protein species generated by the protein unfolding activity of Hsp104 (41).

### Chaperone binding to aggregates generated in the presence of As(III) is impaired

**Figure 6:**
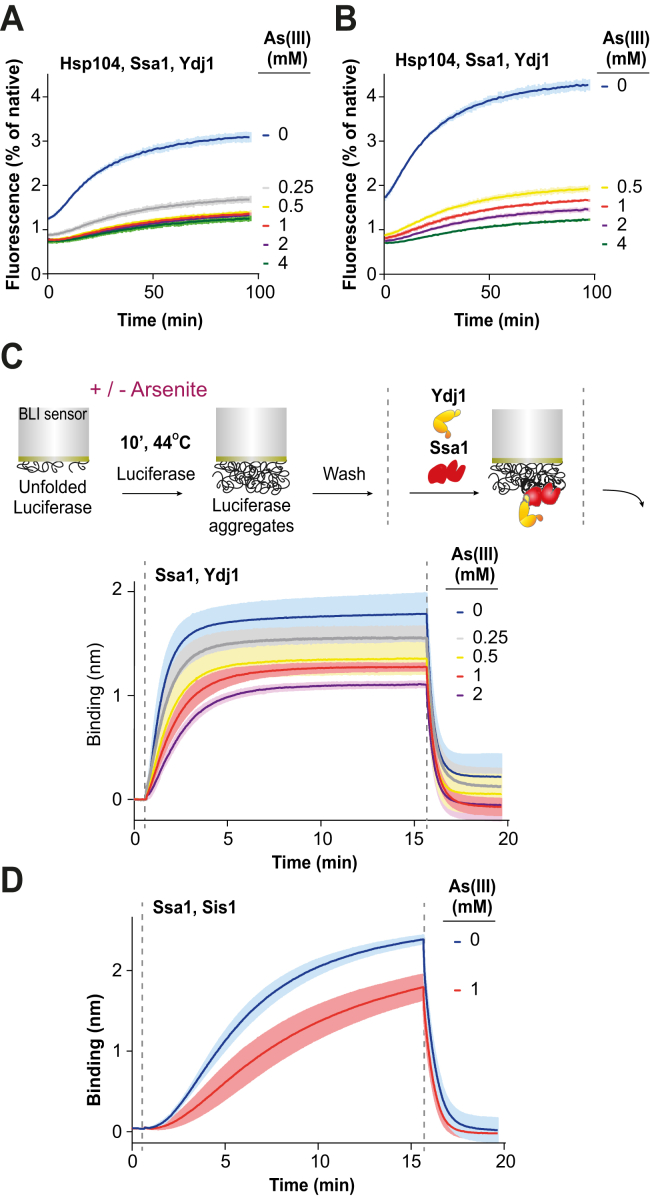
**The presence of As(III) during aggregation inhibits chaperone-mediated protein recovery.***A*, disaggregation and refolding of heat-aggregated GFPuv (0.3 μM) by Ssa1 (1 μM), Ydj1 (1 μM), and Hsp104 (1 μM) chaperones. As(III) was present at both heat aggregation and disaggregation steps at the indicated concentrations. Shown is mean fluorescence normalized to native GFPuv. Error bars represent SD from three repeats. *B*, disaggregation of aggregated GFPuv by chaperones as in (*A*) with modification: As(III) was present at the indicated concentrations only at the heat-aggregation step. During disaggregation, As(III) was diluted 100-fold. *C*, binding of Ssa1 (1.5 μM) and Ydj1 (1 μM) to Luciferase aggregates. The *upper panel* shows a scheme of the BLI experiment. The chaperone binding and dissociation steps are indicated with *dashed lines*. As(III) was present only at the aggregation step at the indicated concentrations. The plot shows mean values of the BLI signal with SD from three experiments. *D*, binding of Ssa1 (1.5 μM) and Sis1 (1 μM) to Luciferase aggregates, performed as in (*C*). BLI, Bio-Layer Interferometry.

To address how As(III) affects the recovery of proteins from aggregates, we purified chaperones constituting the yeast disaggregating machinery: Hsp104 (disaggregase), Ssa1 (Hsp70), and Ydj1/Sis1 (Hsp40s). Previous *in vitro* studies with the bacterial Hsp70 chaperone system (DnaK, DnaJ, GrpE) demonstrated that As(III) inhibits chaperone-assisted refolding of the denatured and heat-aggregated model protein firefly luciferase (11). With the same experimental setup, we observed a similar degree of inhibition by As(III) of luciferase disaggregation by the yeast Hsp70 (Fig. S2*A*) and by the bichaperone Hsp70-Hsp104 system (Fig. S2*B*). Likewise, when we heat aggregated another protein substrate, GFPuv (a GFP variant optimized for maximal fluorescence when excited by UV light (42)), chaperone-mediated fluorescence recovery was strongly inhibited when As(III) was present throughout the experiment (Fig. 6*A*).

We next investigated the effect of As(III) on each individual step in the disaggregation cycle: substrate aggregation, chaperone binding, disaggregation, and refolding. First, we tested if As(III) modulates aggregation in a way that would hamper subsequent aggregate dissolution and protein recovery. When GFPuv was heat aggregated in the presence of increasing concentrations of As(III) and then diluted into the buffer with chaperones and without As(III), the disaggregation rate was negatively correlated with the As(III) concentration present during aggregation (Fig. 6*B*). This indicates that aggregates generated in the presence of As(III) are worse substrates for chaperones than those formed in its absence. To assess chaperone binding to such aggregates, we used Bio-Layer Interferometry (BLI), a technique that allows monitoring the formation of chaperone-aggregate complexes (43). We heat aggregated luciferase at the tip of a sensor in the presence or absence of As(III). After washing, sensors with aggregates were immersed into buffer containing Ssa1 and Ydj1 and without As(III). We found that Ssa1 and Ydj1 binding to these aggregates was inhibited proportionally to the As(III) concentration present during substrate aggregation (Fig. 6*C*). Similar results were obtained with aggregated GFPuv (Fig. S3*A*).

To test if the inhibition is specific to a particular chaperone, we monitored binding to aggregates sequentially, by Ydj1 followed by Ssa1 (Fig. S3*B*) and by Ydj1-Ssa1 followed by Hsp104 (Fig. S3*C*), and observed inhibition at each step.

As(III) is known to bind to cysteine residues in target proteins and this binding may alter protein function and activity (9). Since Ydj1 contains 11 cysteine residues, As(III) might bind to and inhibit Ydj1 function. To address whether diminished binding of Ssa1 and Hsp104 to aggregates formed in the presence of As(III) is a result of Ydj1 inhibition, we replaced Ydj1 with Sis1 that does not contain any cysteines. Interestingly, with Sis1, both disaggregation (Fig. S4, *A*–*D*) and binding to aggregates formed in the presence of As(III) (Fig. 6*D*) were similarly affected as with Ydj1. We also used the highly hyperactive Hsp104 variant Hsp104-D484K, which allows assessing Hsp104 activity independently of Hsp70 (44). Binding of Hsp104-D484K (Fig. S5, *A* and *B*) and all the other analyzed chaperones to aggregates generated in the presence of 1 mM A(III) was reduced by approximately 30% (Figs. 6, *C*, *D* and S3*A*), suggesting that aggregates formed in the presence of As(III) are characterized by limited availability of different chaperone-binding sites.

### Hsp70 and Hsp104 chaperone activity is not directly inhibited by As(III)

**Figure 7:**
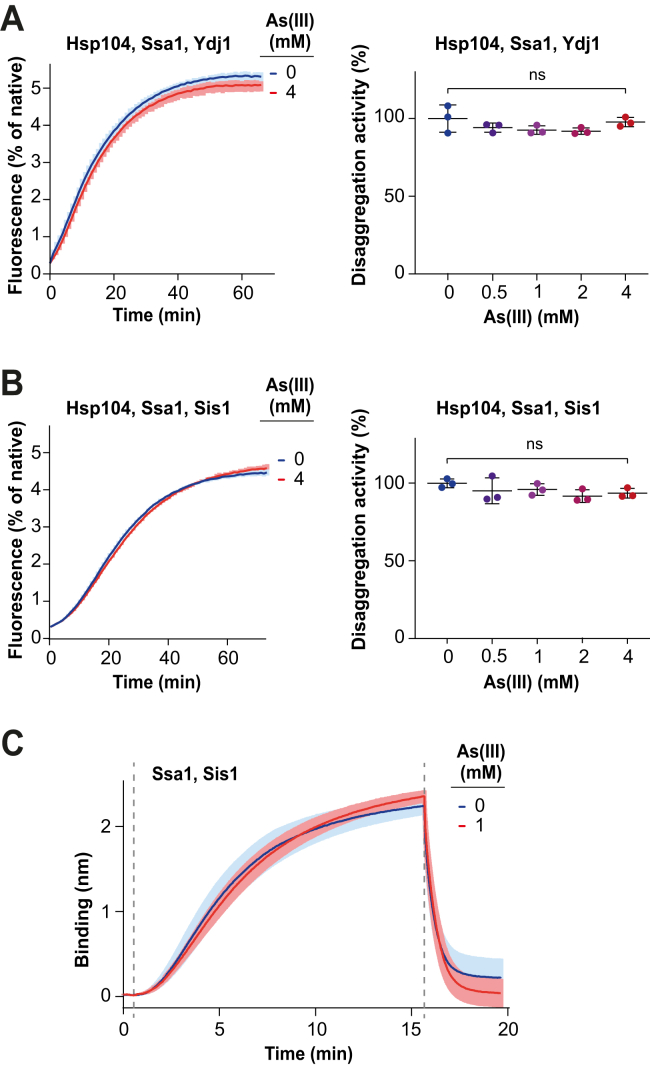
**As(III) does not directly inhibit chaperones involved in disaggregation.***A* and *B*, *left panels*: cfSGFP (0.3 μM) was heat aggregated in the absence of As(III) and disaggregated by Hsp104 (1 μM), Ssa1 (1 μM) with Ydj1 (1 μM) (*A*), or Sis1 (1 μM) (*B*) in the absence or presence of 4 mM As(III). Shown is mean fluorescence normalized to native cfSGFP with SD from three experiments. Right panels: Disaggregation activity of Hsp104-Ssa1-Ydj1 (*A*) and Hsp104-Ssa1-Sis1 (*B*), calculated from the maximal slopes of fluorescence curves shown in left and analogous experiments performed at the indicated As(III) concentrations and normalized to the activity of each chaperone system at 0 mM As(III). Error bars represent SD from three repeats (unpaired, two-tailed Student’s *t* test, ns, nonsignificant *p* > 0.05). *C*, binding of Ssa1 (1.5 μM) and Sis1 (1 μM) to aggregated Luciferase. As(III) was present only at the binding and dissociation steps at 1 mM (*red*) or was absent (*blue*). Shown is mean BLI signal with SD from three repeats. BLI, Bio-Layer Interferometry.

The presence of cysteine residues in Hsp104 (6 cysteines), Ssa1 (3 cysteines), and Ydj1 (11 cysteines) make them potential targets for As(III) binding and inhibition. To address whether As(III) directly inhibits the activity of yeast chaperones, we used cysteine-free SGFP (cfSGFP) (45) as a protein substrate, as its folding should not be influenced by As(III) due to the absence of thiol groups. Interestingly, when cfSGFP was heat aggregated without As(III) and disaggregated by Ydj1-Ssa1-Hsp104 or Sis1-Ssa1-Hsp104 in the presence of increasing As(III) concentration, the rate of fluorescence recovery did not change significantly up to 4 mM As(III) (Fig. 7, *A* and *B*). This observation suggests two things. Firstly, the finding that the defect in chaperone-mediated refolding of the model protein GFPuv (Fig. 6) is abrogated in the cysteine-free version of the substrate cfSGFP (Fig. 7) strongly suggests that the proteotoxic effect of As(III) is due to direct modification of cysteines in non-native target proteins. Secondly, our data also suggest that the activity of the tested chaperones is not affected by As(III). To corroborate this, we performed a BLI experiment with luciferase aggregates generated without As(III) and with the metalloid present only at the chaperone-binding step. This assay could not be performed with Ydj1, which, contrary to Sis1, requires a thiol-containing reducing agent to avoid unspecific interaction with the sensor. Consistent with the result aforementioned, the presence of 1 mM As(III) did not diminish binding of Sis1-Ssa1 (Fig. 7*C*) or Hsp104-D484K (Fig. S5*C*) to the sensor-bound luciferase aggregates. This implies that As(III) does not inactivate the yeast disaggregating machinery by direct binding to the chaperones. Instead, As(III) might inhibit disaggregation by affecting aggregate structure in a way that aggregate-trapped polypeptides are less chaperone exposed and harder to extract from the main body. To explore the latter possibility, we tested if As(III) presence during aggregation hampers subsequent aggregate dissolution by the detergent SDS. The thickness of aggregates at the surface of the BLI sensor was not affected by As(III) presence during their formation (Fig. S6). However, upon subsequent aggregate incubation with 50 mM SDS, their thickness was reduced and the remaining aggregate layer was significantly thicker when As(III) had been present at the aggregation step (Fig. S6). This suggests that As(III) makes aggregating proteins harder to solubilize, regardless of whether by a chaperone or a chemical agent.

### *De novo* protein synthesis is required for efficient aggregate clearance

**Figure 8:**
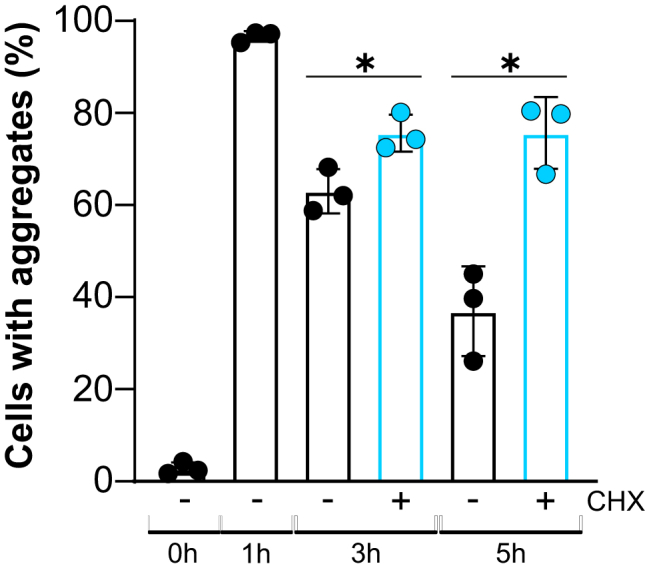
**Protein synthesis is required for efficient aggregate clearance.** Hsp104-GFP distribution was scored in WT cells by fluorescence microscopy before and after exposure to 0.5 mM A(III). After 1 h of exposure, the cell culture was divided where one half was treated with 0.1 mg/ml of cycloheximide (CHX) and the other half was left untreated. The fraction of cells containing aggregates/Hsp104-GFP foci was determined by visual inspection of 318 to 516 cells per condition and time point. Data are expressed as mean ± SD from three independent biological replicates. ∗ indicates a significant difference (*p* < 0.05) compared with cells without CHX (unpaired, two-tailed Student’s *t* test).

Our data aforementioned show that aggregate clearance during As(III) stress depends predominantly on the UPS but also on Hsp104-Hsp70 chaperones and autophagy. Since exposure to As(III) increases the abundance of components of the aforementioned PQC systems (11, 14, 15), we tested whether such increase is important for aggregate clearance. Cells expressing Hsp104-GFP were exposed to As(III), and after 1 h the culture was divided where the protein synthesis inhibitor cycloheximide (CHX) was added to one half of the culture. The addition of CHX strongly attenuated clearance and the fraction of cells with aggregates remained high throughout the time course of the experiment (Fig. 8). Hence, *de novo* protein synthesis is required for efficient aggregate clearance during As(III) exposure.

## Discussion

Arsenic is a global health hazard and a risk factor for pathological conditions associated with protein misfolding and aggregation, including neurodegeneration and cancer (4, 5, 6, 7). Despite of this, our understanding of the underlying mechanisms and cellular responses is limited. Here, we provided novel insights into mechanisms that safeguard proteostasis and cell growth during As(III) exposure.

Chaperone-mediated protein folding and UPS-dependent protein degradation require ATP, and a failure in maintaining high ATP levels was recently shown to result in increased protein aggregation (20). Thus, As(III) could potentially cause protein aggregation or hamper aggregate clearance by ATP depletion. Here, we showed that exponentially growing yeast cells maintain ATP levels during As(III) stress to ensure proteostasis and growth: As(III) concentrations that caused substantial protein aggregation did not impact cellular ATP levels *in vivo*, whereas mutants with reduced intracellular ATP levels (*snf1Δ*, *adk1Δ*) were clearance defective and grew poorly during As(III) stress (Fig. 1). Hence, actively growing cells appear to have sufficient ATP available to power PQC during As(III) stress. In contrast, exposing glucose-starved cells to As(III) resulted in rapid ATP depletion (Fig. 1), and it is reasonable to assume a rapid collapse of proteostasis and reduced cell viability under these conditions.

Several lines of evidence point to the UPS being the main pathway that clears aggregates generated during As(III) exposure. As(III)-treated cells accumulate aggregated proteins with K48-linked Ub chains (Fig. 2), the abundance of proteasomal components and proteasomal activity increases (11, 15), *rpn4Δ* cells have lower proteasomal activity (11) and increased amounts of aggregated proteins with K48-linked Ub chains (Fig. 2), and *rpn4Δ* is As(III) sensitive (11, 15) (Fig. 2). Similarly, chemical inhibition of proteasomal activity affected clearance (Fig. 2), and several mutants defective in proteasomal function and Ub-mediated protein degradation, including *pre1-1 pre4-1* cells (Fig. 2), have been shown to accumulate aggregates and to be As(III) sensitive (11, 13, 46). Conversely, *ubr2Δ* cells that have enhanced UPS activity (30), showed improved clearance and As(III) resistance (Fig. 2).

Studies in yeast have shown that autophagy is activated (as measured by Atg8-GFP fusion cleavage) after 2 to 4 h of exposure to high concentrations of As(III) (15) and that several autophagy-related mutants accumulate more aggregates than WT cells (13). The mutants in the autophagy-vacuole pathway tested here (*atg1Δ*, *atg8Δ, pep4Δ*) showed a delay in aggregate clearance (Fig. 3), suggesting that autophagy targets As(III)-aggregated proteins for vacuolar degradation. While these mutants were not particularly As(III) sensitive (Fig. 3), additional deletion of *ATG8* in *rpn4Δ* cells (*atg8Δ rpn4Δ*) produced a strong synthetic growth defect on As(III) (15). Altogether, these data indicate that the autophagy-vacuole pathway and UPS each contribute to clearance and resistance and that the autophagy-vacuole pathway is less prominent than the UPS. Whether the UPS and autophagy pathways are differently regulated during As(III) stress or whether the aggregates targeted have distinct properties, remains to be investigated.

Using genetic and biochemical approaches, we showed that chaperone-mediated disaggregation contributes to aggregate clearance (Fig. 4). The observations that Hsp104, Ssa1, and Ydj1 cosediment with aggregated proteins during As(III) stress *in vivo* (11) and that Hsp104, Ssa1/Ssa2, and Ydj1 are required for aggregate clearance (Fig. 4), suggest that these chaperones functionally associate with and actively engage in the disaggregation and refolding (35) and/or the degradation (47) of substrate proteins. The cellular concentration of molecular chaperones, including Hsp104, increases during As(III) exposure (11, 13, 14), probably to meet the increased demand for protein folding and disaggregation. We were therefore surprised to find that Hsp104 overexpression was toxic in the presence of this metalloid (Fig. 5). This toxicity was suppressed by GuHCl, raising the possibility that high protein-unfolding activity promiscuously targets native proteins resulting in toxic protein species (41, 43, 44). This might be particularly problematic when cells experience proteotoxic stress caused by As(III). Consistently, the toxicity of potentiated Hsp104-A503V was exacerbated in the presence of As(III) and accompanied with increased protein aggregation. Collectively, these findings suggest that Hsp104 activity must be under strict control during proteotoxic stress, tuned to the cellular need for protein refolding or degradation if the protein damage is irreversible.

As(III) may inhibit protein folding by binding to nascent polypeptide chains and thereby prevent formation of the native protein structure (8, 11). We showed here that the defect in chaperone-mediated refolding of the model protein GFPuv was abrogated in the cysteine-free version of the substrate cfSGFP (Figs. 6, and 7). This finding provides strong support for the notion that the proteotoxic effect of As(III) is primarily caused by direct modification of cysteines in non-native target proteins. As(III) may also target molecular chaperones and inhibit chaperone-assisted protein folding (8, 11). Here, we showed that Hsp104 and the Hsp70 system are not susceptible to As(III) *in vitro* (Fig. 7), even though these chaperones contain multiple cysteine residues. The disaggregation activity was unaffected throughout an hour-long experiment even at 4 mM A(III), suggesting durable insensitivity. This is in contrast to the TRiC chaperonin (48) and many studied thiol-containing enzymes (11, 49, 50, 51) that show gradual or immediate decline in activity at much lower As(III) concentrations. However, since As(III) can target nascent proteins for aggregation (11), we cannot exclude a negative influence of As(III) on chaperones during their synthesis or folding.

Despite the lack of direct inhibition of the chaperones, our *in vitro* data revealed that As(III) does impair protein disaggregation. When unfolded proteins aggregated in the presence of As(III), chaperone interaction with the aggregated substrates and the resultant protein reactivation were reduced, even when As(III) was absent in the latter steps (Fig. 6). Thus, the previously observed failure of yeast cells to clear heat-induced aggregates in the presence of As(III) *in vivo* (11) could be a result of reduced ability of chaperones to bind to aggregated substrates and to solubilize them, rather than by inhibition of chaperone activity. Reduced chaperone binding to aggregates may also explain why aggregates were detected for a longer time in As(III)-exposed cells using the biochemical isolation method (Fig. 2*D*) *versus* GFP-tagged chaperones (Figs. 1*D* and 2*A*). Hence, the dynamics of aggregate formation and clearance *in vivo* might be underestimated using GFP-tagged chaperones as marker proteins. The fact that the observed inhibition is not chaperone specific suggests a general change in aggregate properties in the presence of As(III), which may also limit their processing by other PQC systems. We hypothesize that As(III), which interacts with up to three thiols of the polypeptides trapped in aggregates, may act as a noncovalent crosslinker, limiting their disentanglement. This would explain the reduced solubilization of aggregates that had been generated in the presence of As(III) by SDS (Fig. S6). Consistently, previous *in vitro* studies indicated that As(III) binding can influence the structure of aggregated model proteins. As(III) interacted with and modulated the amyloid fiber structure of the Parkinson’s disease–associated protein α-synuclein (18) and monomethylated arsenite (monomethylarsenous acid) induced the formation of amyloid-like fibrils of bovine pancreatic ribonuclease A (52). Constrained dynamics of polypeptides within the aggregate might limit the ability of chaperones to penetrate the surface and to access their binding sites. This would be in line with the previously observed inhibition of Hsp70 system binding to chemically crosslinked aggregates (53).

The impaired capacity to bind to aggregates might also explain why Hsp104 is largely dispensable for growth and survival during As(III) stress (Fig. 4) (54). Earlier data suggested that intracellular As(III) is predominantly protein bound during acute stress (17). Hence, survival might be linked to the acute phase when target proteins are vulnerable for As(III) interactions and the As(III)-containing aggregates are not (fully) accessible for Hsp104-mediated disaggregation. These aggregates are probably marked by Ub for subsequent proteasomal degradation or targeted by the autophagy pathway. The dispensability of Hsp104 for survival under As(III) stress is in sharp contrast to the importance of chaperone-mediated protein rescue for thermotolerance of yeast and bacterial cells (55, 56). Although our data indicate that As(III) can influence the structure of aggregated model proteins, more work is required to firmly establish whether As(III)-induced aggregates have distinct properties, particularly in the context of their *in vivo* toxicity. Likewise, it will be important to elucidate whether aggregates formed in the presence of As(III) are processed differently from other aggregates. To this end, we recently identified genes and processes that impinge on proteostasis during As(III) stress (13). It remains to be determined whether the identified factors act specifically on As(III)-induced protein aggregates or whether they represent general proteotoxic stress factors.

Protein misfolding and aggregation is not the only mechanism by which As(III) causes toxicity but acts in parallel with other well-described toxicity mechanisms such as oxidative stress–induced damage to DNA, lipids, and proteins, inhibition of DNA repair, and disruption of enzyme function (5, 8, 9). Indeed, we noted that the As(III) sensitivity of a given mutant was not always correlated with aggregate levels, as previously observed (13). The lack of correlation might be a result of a specific function of a gene product in an arsenic resistance pathway that is not linked to protein misfolding and aggregation. Alternatively, the gene product might affect multiple processes that impinge on proteostasis. For example, the lack of sensitivity of *hsp104Δ versus* the sensitivity of *ydj1Δ* (Fig. 4) might be a consequence of Hsp104 targeting aggregated proteins to refolding, whereas Ydj1 is involved in protein degradation in addition to its role in protein refolding (41, 47). Like Ydj1, Ssa1, and Ssa2 are involved in protein degradation in addition to protein refolding; but, in contrast to *ydj1Δ*, *ssa1Δ ssa2Δ* cells were not As(III) sensitive (Fig. 4). Depletion of Ssa is accompanied by decreased translation in yeast (57). Since decreased translation protects cells from As(III) toxicity (11, 13, 15), the lack of sensitivity of *ssa1Δ ssa2Δ* cells might be a consequence of decreased translation in this mutant.

Lastly, we showed that inhibition of *de novo* protein synthesis strongly impaired aggregate clearance during As(III) stress (Fig. 8). This finding suggests that increased abundance of the PQC systems is necessary for clearance and illustrates the dilemma cells face during proteotoxic stress that targets nascent protein folding: by inhibiting translation, as observed in response to As(III) (11, 13, 15), cells can reduce the amounts of nascent proteins that aggregate; however, by doing so, they also prevent efficient clearance of these potentially toxic aggregates. Thus, cells must perform a delicate balancing act between the need to prevent aggregation and the need to deal with the consequences of aggregation.

## Experimental procedures

### Yeast strains, plasmids, and culturing conditions

*S. cerevisiae* strains used in this study (Table S1) are based on BY4741 (58), the yeast deletion collection (59), and WCG4 (60). Double mutants were generated by crossing haploid single mutants using standard procedures. To create the *hsp104Δ rpn4Δ* double mutant, the hygromycin selection cassette was amplified *via* PCR from the vector pFA6-hphNT1 (61) with primers carrying 50 bp of homology on either side to the *RPN4* gene. The resulting PCR product was purified and transformed into the *hsp104Δ* strain followed by selection of colonies on YPD plates containing 200 μg/ml of hygromycin. All double mutants were confirmed by PCR. Yeast cells were grown in rich YPD medium (1% yeast extract, 2% peptone, 2% glucose) or in minimal synthetic complete (SC) medium (0.67% yeast nitrogen base) supplemented with auxotrophic requirements and 2% glucose or 2% galactose as a carbon source. Growth in liquid cultures was measured by optical density at 600 nm and growth assays on solid agar were carried out as previously described (62) with sodium arsenite (NaAsO_2_) (Sigma–Aldrich) added to the cultures at the indicated concentrations. The plasmids used have been described previously and include Sis1-GFP (25), Hsp104, and potentiated Hsp104-A503V behind the *GAL1* promoter (40).

### Measurements of intracellular ATP concentration

Measurements of the average intracellular ATP concentration were made by first quenching the cells with boiling buffered ethanol and subsequently extracting the metabolites as described previously (63). The ATP concentration in the extract was then measured using a Biaffin ATP luciferase bioluminescence kit. The sample (standard or extract) was prepared as recommended by the manufacturer and poured into a 0.4 ml quartz cuvette. The cuvette with the sample was then placed in a temperature-controlled cuvette holder (Quantum Northwest) mounted in a SPEX Fluorolog spectrofluorometer (Edison). The spectrofluorometer was operated in the dark mode and the temperature of the sample was maintained at 25 °C. Bioluminescence from oxyluciferin was measured at 560 nm and recorded for a period of 150 to 300 s. The luminescence signals obtained from the extracts were converted to ATP concentration using a standard curve prepared each day of measurement. Next, the protein content of the extract was determined using the Bradford method. Intracellular ATP concentration was then calculated from the ATP concentration determined in the extract and the corresponding protein concentration assuming an intracellular volume of 3.75 μl per mg protein (64). All ATP and protein measurements were performed in triplicate. All reagents including the Bradford reagent for protein determination and the Biaffin kit for ATP determination were purchased from Merck.

### Fluorescence microscopy

Yeast cells expressing Hsp104–GFP or Sis1–GFP fusion proteins were grown to mid-log phase in SC medium and exposed to 0.5 mM A(III). Where indicated, 3 mM guanidinium hydrochloride (CH_5_N_3_ HCI) (Sigma–Aldrich), 0.1 mg/ml of CHX (Sigma–Aldrich), or 100 μM MG132 (AH Diagnostic) were added to the cell cultures. At the indicated time points, cell samples were fixed with formaldehyde for 30 min at room temperature (RT) and washed with PBS. The GFP signals were observed using a Zeiss Axiovert 200 M (Carl Zeiss MicroImaging) fluorescence microscope equipped with Plan-Apochromat 1.40 objectives and appropriate fluorescence light filter sets. Images were taken with a digital camera (AxioCamMR3) and processed with Zeiss Zen software. To quantify protein aggregation, the total fraction of cells with aggregates (Hsp104–GFP or Sis1-GFP foci) was determined using ImageJ-Fiji software (https://imagej.net/software/fiji/).

### Protein aggregate isolation and analysis

Protein aggregates were isolated as described previously (65). Briefly, cells were grown to log phase in SC medium and exposed to 0.5 mM A(III). At the indicated time points, 50 ml of cell culture was harvested by centrifugation, washed, and resuspended in 300 μl of lysis buffer (50 mM potassium phosphate buffer pH 7, 1 mM EDTA, 5% glycerol, 1 mM PMSF, and EDTA-free complete protease inhibitor cocktail from Roche Diagnostics). Cells were lysed during incubation (30 °C for 30 min) with 100 μl lyticase (10 mg/ml, Sigma–Aldrich), disrupted by sonication (Sonifier 150, Branson, 8 × 5 s, 50% amplitude), and the samples were adjusted to equal protein concentrations before the isolation of protein aggregates. The insoluble fractions were isolated by centrifugation and resuspended in detergent washes (lysis buffer with 2% NP-40 (Sigma–Aldrich)) by sonication (4 × 5 s, 50% amplitude). Samples of total protein lysate and insoluble protein aggregates were resuspended in 2× SDS loading buffer (125 mM Tris–HCI pH 6.7, 6% SDS, 2% glycerol, 10% β-mercaptoethanol, and Bromophenol blue) and boiled for 5 min at 95 °C. Protein samples were loaded on 10% TGX Stain-Free Gels and visualized using Chemidoc (both from Bio-Rad) with UV-activation. For Western blot analysis, protein extracts were transferred to a polyvinylidene difluoride membrane (Bio-Rad) according to the manufacturer’s protocol. Membranes were blocked with blocking buffer (5% nonfat dry milk in Tris-buffered saline [TBS] containing 0.05% Tween 20) for 1 h at RT, incubated overnight at 4 °C with the primary antibody (anti-polyUb K48 linkage at 1:2000 dilution (rabbit, ab1900061, Abcam)), washed with TBS-T, followed by incubation for 2 h at RT with the secondary antibody (Starbright700 anti-rabbit-lgG at 1:5000 dilution (10000068187, Bio-Rad)). Membranes were washed with TBS-T and the signal was detected using Chemidoc (Bio-Rad). Images were analyzed using ImageJ. To quantify the levels of protein aggregation, the signal in the aggregate fraction was first normalized to the signal in the corresponding total lysate input and then to the total signal over all samples to allow comparison between gels. To quantify K48 Ub levels in the aggregate fraction, the signal in the aggregate fraction was first normalized to the corresponding total lysate input and then normalized to the total signal over all samples to allow comparison between blots.

### Proteins

We used previously published protocols to purify Hsp104 (44), Ssa1 (66), Sis1 (67), Ydj1 (44), His-tagged Luciferase (43), and GFPuv (68). Creatine kinase was purchased from Sigma–Aldrich (10127566001). Untagged Luciferase was purchased from Promega (E1701). cfSGFP gene (45) was synthesized and cloned by Genescript to pET3a plasmid and expressed in the *Escherichia coli* BL21(DE3) codon+ strain. The soluble fraction of the lysate was mixed 1:1 with 96% ethanol and centrifuged. Supernatant was loaded on a Q-Sepharose fast flow (GE Healthcare Bio-Sciences AB) column in 40 mM Tris–HCl, pH 7.5, 10% glycerol, 50 mM NaCl, and eluted with 40 mM Tris–HCl, pH 7.5, 10% glycerol, 300 mM NaCl. Fractions with cfSGFP were dialyzed into 40 mM Tris–HCl, pH 7.5, 20% glycerol, 50 mM NaCl, heated for 15 min at 73.2 °C, and rapidly cooled down to precipitate protein impurities. The soluble fraction was stored at −80 °C. Protein concentrations are indicated in the figures. All the protein concentrations refer to monomer.

### GFP disaggregation

Heat-aggregated GFPuv and cfSGFP renaturation assays were performed as previously described (68) with modifications. The buffer contained sodium arsenite (Sigma–Aldrich, S7400) when indicated in the Figures, and it did not contain reducing agents. GFPuv was aggregated at 85 °C and cfSGFP at 77.8 °C for 15 min. Fluorescence was measured using the Beckman Coulter DTX 880 Plate Reader. Statistical analysis was performed using GraphPad Prism software (GraphPad Software Inc).

### BLI experiments

Aggregate-binding experiments were performed as previously described (43, 53) with modifications. The hydrated Ni-NTA sensor (ForteBio) was incubated in buffer A (25 mM Hepes–KOH, pH 8, 15 mM magnesium acetate, 75 mM KCl) with 6 M urea and 8.2 μM His-tagged Luciferase for 10 min and washed with buffer A for 5 min. Next, the sensor was transferred to buffer A containing 1.6 μM of the native His-tagged Luciferase and sodium arsenite at the concentrations indicated in the Figures. After incubation for 10 min at 44 °C, the sensor was equilibrated for 10 min with buffer A. As(III) did not affect Luciferase binding to the Ni-NTA sensor nor change the final aggregate thickness. The baseline, chaperone binding, and dissociation steps were performed in buffer A with 10 mM ATP and the indicated concentrations of sodium arsenite. Experimental steps involving Ydj1 were performed in the presence of 2 mM DTT, which was necessary to prevent an unspecific interaction with the sensor. Binding of Hsp104 D484K was performed in the presence of an ATP regeneration system comprising 1.2 μM creatine kinase and 20 mM creatine phosphate. The BLI signal was detected using the BLItz and Octet K2 instruments (ForteBio). If not stated otherwise, all the steps were carried out at 25 °C.

## Data availability

All the data described are contained within the article.

## Supporting information

This article contains supporting information (11, 38, 60, 69, 70).

## Conflict of interest

The authors declare that they have no conflicts of interest with the contents of this article.

## References

[1] Hipp M.S., Kasturi P., Hartl F.U. (2019). The proteostasis network and its decline in ageing. Nat. Rev. Mol. Cell Biol..

[2] Sala A.J., Bott L.C., Morimoto R.I. (2017). Shaping proteostasis at the cellular, tissue, and organismal level. J. Cell Biol..

[3] Podgorski J., Berg M. (2020). Global threat of arsenic in groundwater. Science.

[4] Naujokas M.F., Anderson B., Ahsan H., Aposhian H.V., Graziano J.H., Thompson C. (2013). The broad scope of health effects from chronic arsenic exposure: update on a worldwide public health problem. Environ. Health Perspect..

[5] Zhou Q., Xi S. (2018). A review on arsenic carcinogenesis: epidemiology, metabolism, genotoxicity and epigenetic changes. Regul. Toxicol. Pharmacol..

[6] Raj K., Kaur P., Gupta G.D., Singh S. (2021). Metals associated neurodegeneration in Parkinson's disease: insight to physiological, pathological mechanisms and management. Neurosci. Lett..

[7] Chin-Chan M., Navarro-Yepes J., Quintanilla-Vega B. (2015). Environmental pollutants as risk factors for neurodegenerative disorders: Alzheimer and Parkinson diseases. Front Cell Neurosci..

[8] Tamás M.J., Sharma K.S., Ibstedt S., Jacobson T., Christen P. (2014). Heavy metals and metalloids as a cause for protein misfolding and aggregation. Biomolecules.

[9] Shen S., Li X.F., Cullen W.R., Weinfeld M., Le X.C. (2013). Arsenic binding to proteins. Chem. Rev..

[10] Tam L.M., Wang Y. (2020). Arsenic exposure and compromised protein quality control. Chem. Res. Toxicol..

[11] Jacobson T., Navarrete C., Sharma S.K., Sideri T.C., Ibstedt S., Priya S. (2012). Arsenite interferes with protein folding and triggers formation of protein aggregates in yeast. J. Cell Sci..

[12] Ibstedt S., Sideri T.C., Grant C.M., Tamás M.J. (2014). Global analysis of protein aggregation in yeast during physiological conditions and arsenite stress. Biol. Open.

[13] Andersson S., Romero A., Rodrigues J.I., Hua S., Hao X., Jacobson T. (2021). Genome-wide imaging screen uncovers molecular determinants of arsenite-induced protein aggregation and toxicity. J. Cell Sci..

[14] Thorsen M., Lagniel G., Kristiansson E., Junot C., Nerman O., Labarre J. (2007). Quantitative transcriptome, proteome, and sulfur metabolite profiling of the *Saccharomyces cerevisiae* response to arsenite. Physiol. Genomics.

[15] Guerra-Moreno A., Isasa M., Bhanu M.K., Waterman D.P., Eapen V.V., Gygi S.P. (2015). Proteomic analysis identifies ribosome reduction as an effective proteotoxic stress response. J. Biol. Chem..

[16] Haugen A.C., Kelley R., Collins J.B., Tucker C.J., Deng C., Afshari C.A. (2004). Integrating phenotypic and expression profiles to map arsenic-response networks. Genome Biol..

[17] Talemi S.R., Jacobson T., Garla V., Navarrete C., Wagner A., Tamás M.J. (2014). Mathematical modelling of arsenic transport, distribution and detoxification processes in yeast. Mol. Microbiol..

[18] Lorentzon E., Horvath I., Kumar R., Rodrigues J.I., Tamás M.J., Wittung-Stafshede P. (2021). Effects of the toxic metals arsenite and cadmium on alpha-synuclein aggregation *in vitro* and in cells. Int. J. Mol. Sci..

[19] Sathyanarayanan U., Musa M., Bou Dib P., Raimundo N., Milosevic I., Krisko A. (2020). ATP hydrolysis by yeast Hsp104 determines protein aggregate dissolution and size *in vivo*. Nat. Commun..

[20] Takaine M., Imamura H., Yoshida S. (2022). High and stable ATP levels prevent aberrant intracellular protein aggregation in yeast. eLife.

[21] Patel A., Malinovska L., Saha S., Wang J., Alberti S., Krishnan Y. (2017). ATP as a biological hydrotrope. Science.

[22] Xu L., Bretscher A. (2014). Rapid glucose depletion immobilizes active myosin V on stabilized actin cables. Curr. Biol..

[23] Zhang H.N., Yang L., Ling J.Y., Czajkowsky D.M., Wang J.F., Zhang X.W. (2015). Systematic identification of arsenic-binding proteins reveals that hexokinase-2 is inhibited by arsenic. Proc. Natl. Acad. Sci. U. S. A..

[24] Yan W., Craig E.A. (1999). The glycine-phenylalanine-rich region determines the specificity of the yeast Hsp40 Sis1. Mol. Cell Biol..

[25] Malinovska L., Kroschwald S., Munder M.C., Richter D., Alberti S. (2012). Molecular chaperones and stress-inducible protein-sorting factors coordinate the spatiotemporal distribution of protein aggregates. Mol. Biol. Cell.

[26] Park S.H., Kukushkin Y., Gupta R., Chen T., Konagai A., Hipp M.S. (2013). PolyQ proteins interfere with nuclear degradation of cytosolic proteins by sequestering the Sis1p chaperone. Cell.

[27] Choe Y.J., Park S.H., Hassemer T., Korner R., Vincenz-Donnelly L., Hayer-Hartl M. (2016). Failure of RQC machinery causes protein aggregation and proteotoxic stress. Nature.

[28] Mannhaupt G., Schnall R., Karpov V., Vetter I., Feldmann H. (1999). Rpn4p acts as a transcription factor by binding to PACE, a nonamer box found upstream of 26S proteasomal and other genes in yeast. FEBS Lett..

[29] Hilt W., Enenkel C., Gruhler A., Singer T., Wolf D.H. (1993). The *PRE4* gene codes for a subunit of the yeast proteasome necessary for peptidylglutamyl-peptide-hydrolyzing activity. Mutations link the proteasome to stress- and ubiquitin-dependent proteolysis. J. Biol. Chem..

[30] Wang L., Mao X., Ju D., Xie Y. (2004). Rpn4 is a physiological substrate of the Ubr2 ubiquitin ligase. J. Biol. Chem..

[31] Yau R., Rape M. (2016). The increasing complexity of the ubiquitin code. Nat. Cell Biol..

[32] Yin Z., Pascual C., Klionsky D.J. (2016). Autophagy: machinery and regulation. Microb. Cell.

[33] Ammerer G., Hunter C.P., Rothman J.H., Saari G.C., Valls L.A., Stevens T.H. (1986). *PEP4* gene of *Saccharomyces cerevisiae* encodes proteinase A, a vacuolar enzyme required for processing of vacuolar precursors. Mol. Cell Biol..

[34] Woolford C.A., Daniels L.B., Park F.J., Jones E.W., Van Arsdell J.N., Innis M.A. (1986). The *PEP4* gene encodes an aspartyl protease implicated in the posttranslational regulation of *Saccharomyces cerevisiae* vacuolar hydrolases. Mol. Cell Biol..

[35] Glover J.R., Lindquist S. (1998). Hsp104, Hsp70, and Hsp40: a novel chaperone system that rescues previously aggregated proteins. Cell.

[36] Ferreira P.C., Ness F., Edwards S.R., Cox B.S., Tuite M.F. (2001). The elimination of the yeast [PSI+] prion by guanidine hydrochloride is the result of Hsp104 inactivation. Mol. Microbiol..

[37] Jung G., Jones G., Masison D.C. (2002). Amino acid residue 184 of yeast Hsp104 chaperone is critical for prion-curing by guanidine, prion propagation, and thermotolerance. Proc. Natl. Acad. Sci. U. S. A..

[38] Lum R., Tkach J.M., Vierling E., Glover J.R. (2004). Evidence for an unfolding/threading mechanism for protein disaggregation by *Saccharomyces cerevisiae* Hsp104. J. Biol. Chem..

[39] Wysocki R., Tamás M.J. (2010). How *Saccharomyces cerevisiae* copes with toxic metals and metalloids. FEMS Microbiol. Rev..

[40] Jackrel M.E., DeSantis M.E., Martinez B.A., Castellano L.M., Stewart R.M., Caldwell K.A. (2014). Potentiated Hsp104 variants antagonize diverse proteotoxic misfolding events. Cell.

[41] den Brave F., Cairo L.V., Jagadeesan C., Ruger-Herreros C., Mogk A., Bukau B. (2020). Chaperone-mediated protein disaggregation triggers proteolytic clearance of intra-nuclear protein inclusions. Cell Rep..

[42] Crameri A., Whitehorn E.A., Tate E., Stemmer W.P. (1996). Improved green fluorescent protein by molecular evolution using DNA shuffling. Nat. Biotechnol..

[43] Chamera T., Klosowska A., Janta A., Wyszkowski H., Obuchowski I., Gumowski K. (2019). Selective hsp70-dependent docking of Hsp104 to protein aggregates protects the cell from the toxicity of the disaggregase. J. Mol. Biol..

[44] Lipinska N., Zietkiewicz S., Sobczak A., Jurczyk A., Potocki W., Morawiec E. (2013). Disruption of ionic interactions between the nucleotide binding domain 1 (NBD1) and middle (M) domain in Hsp100 disaggregase unleashes toxic hyperactivity and partial independence from Hsp70. J. Biol. Chem..

[45] Suzuki T., Arai S., Takeuchi M., Sakurai C., Ebana H., Higashi T. (2012). Development of cysteine-free fluorescent proteins for the oxidative environment. PLoS One.

[46] Hanna J., Waterman D., Isasa M., Elsasser S., Shi Y., Gygi S. (2014). Cuz1/Ynl155w, a zinc-dependent ubiquitin-binding protein, protects cells from metalloid-induced proteotoxicity. J. Biol. Chem..

[47] Breckel C.A., Hochstrasser M. (2021). Ubiquitin ligase redundancy and nuclear-cytoplasmic localization in yeast protein quality control. Biomolecules.

[48] Pan X., Reissman S., Douglas N.R., Huang Z., Yuan D.S., Wang X. (2010). Trivalent arsenic inhibits the functions of chaperonin complex. Genetics.

[49] Lu J., Chew E.H., Holmgren A. (2007). Targeting thioredoxin reductase is a basis for cancer therapy by arsenic trioxide. Proc. Natl. Acad. Sci. U. S. A..

[50] Chouchane S., Snow E.T. (2001). *In vitro* effect of arsenical compounds on glutathione-related enzymes. Chem. Res. Toxicol..

[51] Yoda A., Toyoshima K., Watanabe Y., Onishi N., Hazaka Y., Tsukuda Y. (2008). Arsenic trioxide augments Chk2/p53-mediated apoptosis by inhibiting oncogenic Wip1 phosphatase. J. Biol. Chem..

[52] Ramadan D., Rancy P.C., Nagarkar R.P., Schneider J.P., Thorpe C. (2009). Arsenic(III) species inhibit oxidative protein folding *in vitro*. Biochemistry.

[53] Wyszkowski H., Janta A., Sztangierska W., Obuchowski I., Chamera T., Klosowska A. (2021). Class-specific interactions between Sis1 J-domain protein and Hsp70 chaperone potentiate disaggregation of misfolded proteins. Proc. Natl. Acad. Sci. U. S. A..

[54] Sanchez Y., Taulien J., Borkovich K.A., Lindquist S. (1992). Hsp104 is required for tolerance to many forms of stress. EMBO J..

[55] Sanchez Y., Lindquist S.L. (1990). HSP104 required for induced thermotolerance. Science.

[56] Weibezahn J., Tessarz P., Schlieker C., Zahn R., Maglica Z., Lee S. (2004). Thermotolerance requires refolding of aggregated proteins by substrate translocation through the central pore of ClpB. Cell.

[57] Horton L.E., James P., Craig E.A., Hensold J.O. (2001). The yeast hsp70 homologue Ssa is required for translation and interacts with Sis1 and Pab1 on translating ribosomes. J. Biol. Chem..

[58] Brachmann C.B., Davies A., Cost G.J., Caputo E., Li J., Hieter P. (1998). Designer deletion strains derived from *Saccharomyces cerevisiae* S288C: a useful set of strains and plasmids for PCR-mediated gene disruption and other applications. Yeast.

[59] Giaever G., Chu A.M., Ni L., Connelly C., Riles L., Veronneau S. (2002). Functional profiling of the *Saccharomyces cerevisiae* genome. Nature.

[60] Heinemeyer W., Gruhler A., Mohrle V., Mahe Y., Wolf D.H. (1993). PRE2, highly homologous to the human major histocompatibility complex-linked RING10 gene, codes for a yeast proteasome subunit necessary for chrymotryptic activity and degradation of ubiquitinated proteins. J. Biol. Chem..

[61] Janke C., Magiera M.M., Rathfelder N., Taxis C., Reber S., Maekawa H. (2004). A versatile toolbox for PCR-based tagging of yeast genes: new fluorescent proteins, more markers and promoter substitution cassettes. Yeast.

[62] Wysocki R., Fortier P.K., Maciaszczyk E., Thorsen M., Leduc A., Odhagen A. (2004). Transcriptional activation of metalloid tolerance genes in *Saccharomyces cerevisiae* requires the AP-1-like proteins Yap1p and Yap8p. Mol. Biol. Cell.

[63] Gonzalez B., Francois J., Renaud M. (1997). A rapid and reliable method for metabolite extraction in yeast using boiling buffered ethanol. Yeast.

[64] Richard P., Teusink B., Hemker M.B., Van Dam K., Westerhoff H.V. (1996). Sustained oscillations in free-energy state and hexose phosphates in yeast. Yeast.

[65] Weids A.J., Grant C.M. (2014). The yeast peroxiredoxin Tsa1 protects against protein-aggregate-induced oxidative stress. J. Cell Sci..

[66] Andréasson C., Fiaux J., Rampelt H., Druffel-Augustin S., Bukau B. (2008). Insights into the structural dynamics of the Hsp110-Hsp70 interaction reveal the mechanism for nucleotide exchange activity. Proc. Natl. Acad. Sci. U. S. A..

[67] Shorter J., Lindquist S. (2004). Hsp104 catalyzes formation and elimination of self-replicating Sup35 prion conformers. Science.

[68] Zietkiewicz S., Krzewska J., Liberek K. (2004). Successive and synergistic action of the Hsp70 and Hsp100 chaperones in protein disaggregation. J. Biol. Chem..

[69] Öling D., Eisele F., Kvint K., Nyström T. (2014). Opposing roles of Ubp3-dependent deubiquitination regulate replicative life span and heat resistance. EMBO J..

[70] Gerlinger U.M., Guckel R., Hoffmann M., Wolf D.H., Hilt W. (1997). Yeast cycloheximide-resistant crl mutants are proteasome mutants defective in protein degradation. Mol. Biol. Cell.

