# Supplementary material for: Differential contributions of the proteasome, autophagy, and chaperones to the clearance of arsenite-induced protein aggregates in yeast
Source: J Biol Chem. 2022 Nov 7;298(12):102680. doi: 10.1016/j.jbc.2022.102680 (PMC9723941; doi:10.1016/j.jbc.2022.102680)
Supplement: Supporting information [file mmc1.docx]

Supporting information for:

**Differential contributions of the proteasome, autophagy, and chaperones to the clearance of arsenite-induced protein aggregates in yeast**

**Sansan Hua, Agnieszka Kłosowska, Joana I. Rodrigues, Gabriel Petelski, Lidia Alejo Esquembre, Emma Lorentzon, Lars F. Olsen, Krzysztof Liberek and Markus J. Tamás**

**Material included:**

**Supporting Figures S1 to S6**

**Supporting Experimental procedures for Figures S2 to S5**

**Supporting Table S1**

**Figure S1. A**) Sis1–GFP (upper panel) or Hsp104-GFP (lower panel) localization was monitored in wild type cells by fluorescence microscopy before and after exposure to 0.5 mM As(III). Representative images are shown. **(B)** The aggregates decline faster than the increase in cell number. Shown is the % change in the fraction of cells with aggregates and the increase in optical density (OD) measured at 600 nm at the indicated time-points. Data are expressed as mean ± S.D. from nine independent biological replicates. * indicates a significant difference (*p*<0.05).

**
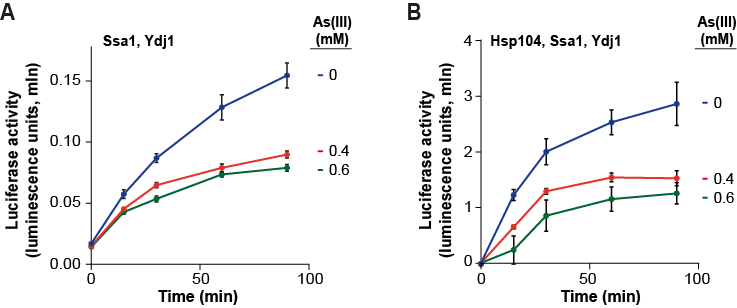
**

**Figure S2.** Disaggregation and refolding of heat-aggregated Luciferase by Ssa1 (1 μM) and Ydj1 (1 μM) **(A)** or by Hsp104 (1 μM), Ssa1 (1 μM) and Ydj1 (1 μM) **(B)** at the indicated As(III) concentrations. Error bars show S.D. from three experiments.


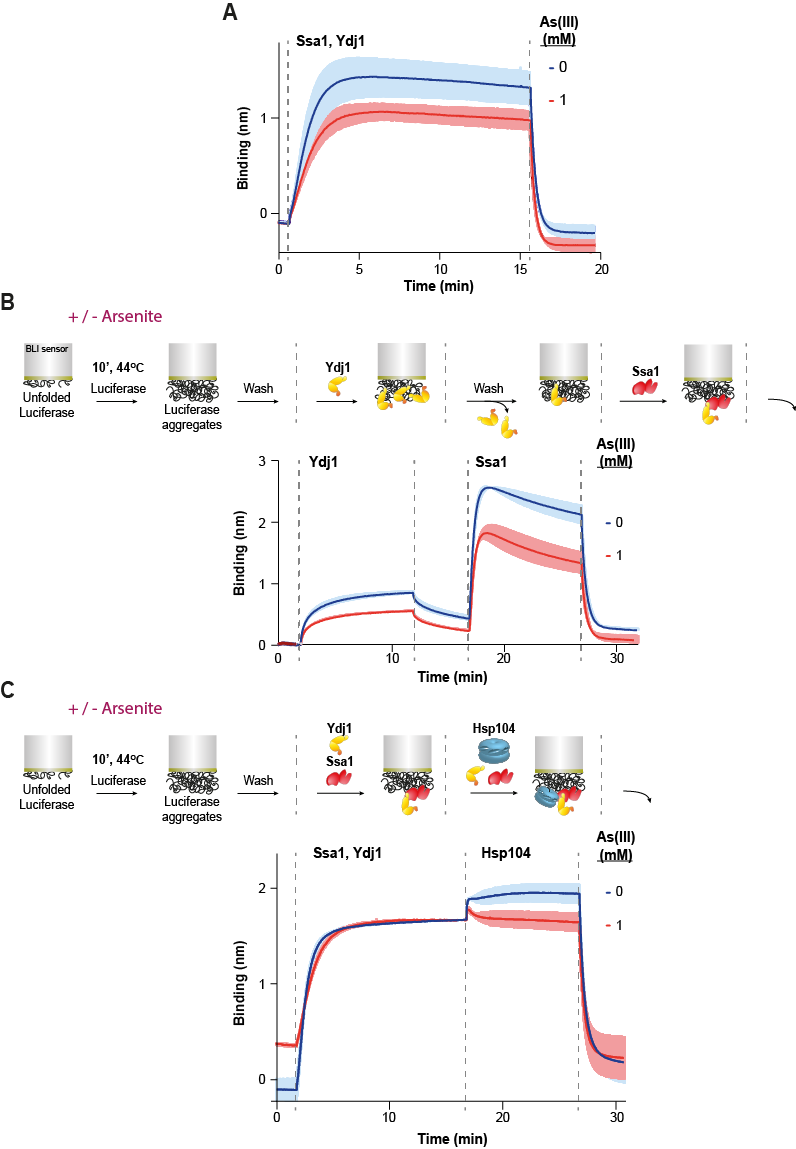


**Figure S3. A)** Binding of Ssa1 (1.5 µM) and Ydj1 (1 µM) to GFPuv aggregates. The chaperone binding and dissociation steps are indicated with dashed lines. 1 mM As(III) was present only at the aggregation step or was absent throughout the experiment, as indicated in the legend. The line shows mean values and shaded outlines show BLI signal from two experiments. **B)** Sequential binding of Ydj1 (2 µM) and Ssa1 (1.5 µM) to Luciferase aggregates, performed according to the scheme in the upper panel. 1 mM As(III) was present only during aggregation. Lower panel shows BLI signal from two repeats as in **A**. **C)** Binding of Ssa1 (1.5 µM) and Ydj1 (1 µM) followed by Hsp104 (2 µM) as in the scheme in the upper panel. As(III) at the indicated concentration was present only during aggregation. The plots were overlaid at the association of Hsp104. Shades represent S.D. from three repeats.

**
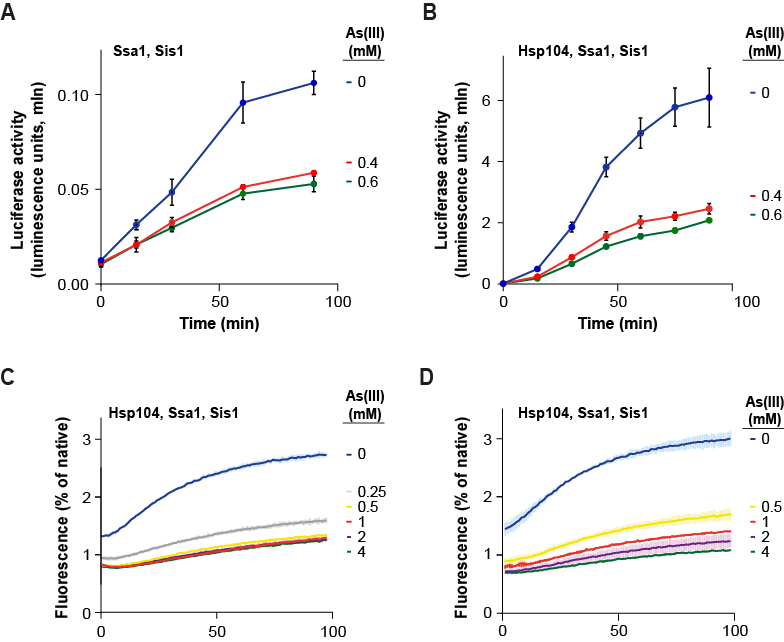
**

**Figure S4.** Disaggregation and refolding of heat-aggregated Luciferase by Ssa1 (1 μM) and Sis1 (1 μM) **(A)** or by Hsp104 (1 μM), Ssa1 (1 μM) and Sis1 (1 μM) **(B)** at the indicated As(III) concentrations. Error bars show S.D. from three experiments. **C)** Disaggregation and refolding of heat-aggregated GFPuv (0.3 µM) by Ssa1 (1 µM), Sis1 (1 µM) and Hsp104 (1 µM) chaperones. As(III) was present at both heat-aggregation and disaggregation steps at the indicated concentrations. Error bars represent S.D. from three repeats. **D)** Disaggregation of aggregated GFPuv by chaperones as in **C**, but with As(III) at the indicated concentrations present only at the heat-aggregation step. During disaggregation, As(III) was diluted 100-fold.

**
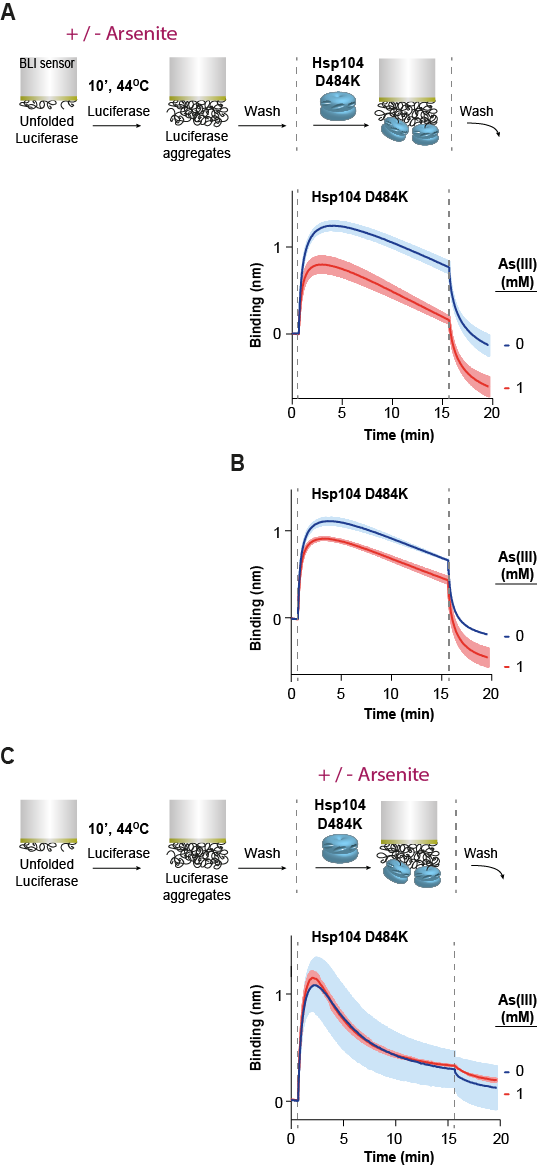
**

**Figure S5.** Binding of Hsp104 D484K (2 µM) to Luciferase **(A)** and GFPuv **(B)** aggregates. 1 mM As(III) was present (red) only at the aggregation step or was absent (blue) throughout the experiment. The plot shows mean values of the BLI signal. Shades indicate S.D. from four **(A)** or values from two experiments **(B)**. **C)** Binding of Hsp104 D484K (2 µM) to Luciferase aggregates with As(III) present only during Hsp104 D484K binding and dissociation. Line represents mean and shaded outlines show BLI signal from two repeats.


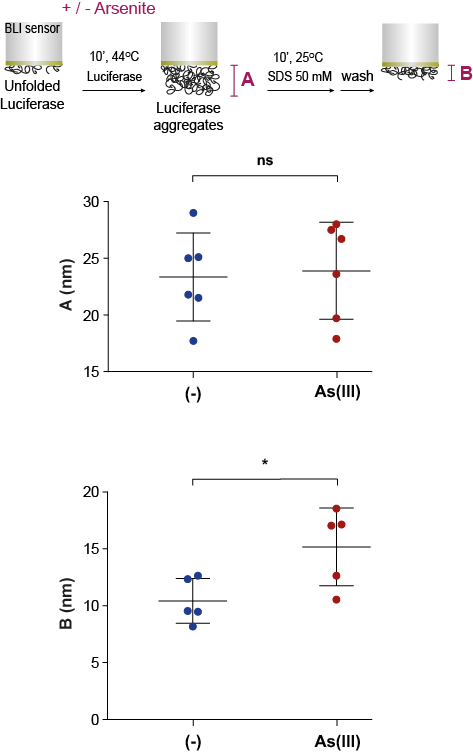


**Figure S6.** Upper panel shows experimental scheme. **A** and **B** indicate the steps, at which aggregate thickness was measured with BLI. As(III) was present only at the aggregation step or was absent throughout the experiment. Middle panel: aggregate thickness after aggregation **(A)**. Lower panel: Aggregate thickness after the incubation of aggregate-covered sensors with 50 mM SDS for 10 min and washing **(B)**. Plotted data points represent aggregates generated in the presence of 4 mM As(III) (red) or without As(III) (blue), with median and S.D. (t-test, * *p*<0.05, ns *p*>0.05).

**Supporting Experimental procedures for Figures S2-S5**

**Luciferase Refolding Assay.**

Luciferase (1.875 mg/mL) was chemically denatured in buffer A (25 mM HEPES-KOH, pH 7.5, 15 mM magnesium acetate, 75 mM KCl) with 6 M urea and incubated at 25°C for 15 min. Subsequently, it was transferred to 48°C, incubated for 10 min, and rapidly diluted 25 times with the buffer without urea. Final luciferase concentration in the reaction was 0.2 µM. Disaggregation was carried out in buffer A with 10 mM ATP and chaperones at the concentrations indicated in the Figures, at 25°C. Luciferase activity was measured using Luciferase Assay Kit (Promega, E1501) with Sirius Luminometer (Berthold).

**Biolayer interferometry (BLI) experiments**

Sensors with GFPuv aggregates were prepared as follows: 12.5 µM of His-tagged GFPuv was incubated in buffer A with 10 M urea at 85°C for 10 min and then incubated with the Ni-NTA sensor for 10 min. After washing with buffer A for 5 min, the sensor was incubated at 85°C for 15 min with 4.2 µM His-tagged GFPuv in buffer A containing the indicated concentrations of sodium arsenite. As(III) did not affect the thickness of the aggregates. After washing, the sensor was incubated in buffer A with 10 mM ATP and chaperones at the concentrations indicated in the Figures. The BLI signal was detected using the BLItz instrument (ForteBio). Unless stated otherwise, all the steps were carried out at 25°C.

| **Table S1.** Yeast strains used. | |  |
| --- | --- | --- |
| **Strain** | **Genotype** | **Reference/Source** |
| BY4741 | *MATa his3Δ1 leu2Δ0 met15Δ0 ura3Δ0* | EUROSCARF |
| Hsp104 GFP | *MATa his3Δ1 leu2Δ0 met15Δ0 ura3Δ0 HSP104-GFP-HIS3-MX6* | EUROSCARF |
| Hsp104-Y662A-GFP | *MATa his3Δ1 leu2Δ0 met15Δ0 ura3Δ0 HSP104 Y662A-GFP-HIS3* | [38] |
| *rpn4Δ* | BY4741 *rpn4Δ::KanMX4* | EUROSCARF |
| *atg1Δ* | BY4741 *atg1Δ::KanMX4* | EUROSCARF |
| *atg8Δ* | BY4741 *atg8Δ::KanMX4* | EUROSCARF |
| *pep4Δ* | BY4741 *pep4Δ::KanMX4* | EUROSCARF |
| *ubr2Δ* | BY4741 *ubr2Δ::KanMX4* | EUROSCARF |
| *ydj1Δ* | BY4741 *ydj1Δ::KanMX4* | EUROSCARF |
| *ssa1Δ ssa2Δ* | BY4741 *ssa1Δ::hph ssa2Δ::kanMX4* | [69] |
| *hsp104Δ* | BY4741 *hsp104Δ::KanMX4* | EUROSCARF |
| *acr3Δ* | BY4741 *acr3Δ::KanMX4* | EUROSCARF |
| *yap8Δ* | BY4741 *yap8Δ::KanMX4* | EUROSCARF |
| *hsp104Δ yap8Δ* | BY4741 *hsp104Δ::KanMX4* *yap8Δ::KanMX4* | this study |
| *hsp104Δ acr3Δ* | BY4741 *hsp104Δ::KanMX4* *acr3Δ::KanMX4* | this study |
| *hsp104Δ rpn4Δ* | BY4741 *hsp104Δ::KanMX4* *rpn4Δ::HygR* | this study |
| Hsp104-GFP *rpn4Δ* | *MATa lys2Δ0 HSP104-GFP-HIS3-MX6 rpn4Δ::KanMX4* | [11] |
| WCG4 | *MATa ura3 leu2-3,112 his3-11,15 CanS Gal+* | [60] |
| YHI29/14 | *MATa ura3 leu2-3,112 his3-11,15 CanS Gal+ pre1-1 pre4-1* | [70] |
| Hsp104-GFP *atg1Δ* | *MATalpha met15Δ0 HSP104-GFP-HIS3-MX6 atg1Δ::KanMX4* | this study |
| Hsp104-GFP *atg8Δ* | *MATa met15Δ0 HSP104-GFP-HIS3-MX6 atg8Δ::KanMX4* | this study |
| Hsp104-GFP *pep4Δ* | *MATa met15Δ0 HSP104-GFP-HIS3-MX6 pep4Δ::KanMX4* | this study |
|  |  |  |
| [11] T. Jacobson, C. Navarrete, S.K. Sharma, T.C. Sideri, S. Ibstedt, S. Priya, C.M. Grant, P. Christen, P. Goloubinoff, M.J. Tamás, Arsenite interferes with protein folding and triggers formation of protein aggregates in yeast, J Cell Sci, 125 (2012) 5073-5083. | | |
| [38] R. Lum, J.M. Tkach, E. Vierling, J.R. Glover, Evidence for an unfolding/threading mechanism for protein disaggregation by *Saccharomyces cerevisiae* Hsp104, J Biol Chem, 279 (2004) 29139-29146. | | |
| [60] Heinemeyer, W., Gruhler, A., Mohrle, V., Mahe, Y., and Wolf, D. H. (1993) PRE2, highly homologous to the human major histocompatibility complex-linked RING10 gene, codes for a yeast proteasome subunit necessary for chrymotryptic activity and degradation of ubiquitinated proteins. J Biol Chem 268, 5115-5120. | | |
| [69] D. Öling, F. Eisele, K. Kvint, T. Nyström, Opposing roles of Ubp3-dependent deubiquitination regulate replicative life span and heat resistance, EMBO J, 33 (2014) 747-761. | | |
| [70] Gerlinger, U. M., Guckel, R., Hoffmann, M., Wolf, D. H., and Hilt, W. (1997) Yeast cycloheximide-resistant crl mutants are proteasome mutants defective in protein degradation. Mol Biol Cell 8, 2487-2499. | | |
